# Real‐world safety and effectiveness of anamorelin for cancer cachexia: Interim analysis of post‐marketing surveillance in Japan

**DOI:** 10.1002/cam4.7170

**Published:** 2024-05-01

**Authors:** Koichi Takayama, Ai Kojima, Chikara Honda, Masahiro Nakayama, Satomi Kanemata, Toshimitsu Endo, Kei Muro

**Affiliations:** ^1^ Department of Pulmonary Medicine, Graduate School of Medical Science Kyoto Prefectural University of Medicine Kyoto Japan; ^2^ Pharmacovigilance Ono Pharmaceutical Co., Ltd. Osaka Japan; ^3^ Medical Affairs, Ono Pharmaceutical Co., Ltd. Osaka Japan; ^4^ Department of Clinical Oncology Aichi Cancer Center Hospital Nagoya Japan

**Keywords:** anamorelin, appetite, body weight, cancer cachexia, clinical practice, post‐marketing surveillance

## Abstract

**Background:**

Anamorelin was approved in Japan in 2021 to treat cancer cachexia associated with non‐small cell lung, gastric, pancreatic, or colorectal cancers. Post‐marketing surveillance is being conducted to evaluate the real‐world safety and effectiveness of anamorelin.

**Methods:**

This prospective, observational surveillance registered all patients who started treatment with anamorelin after April 21, 2021. Hyperglycemia, hepatic impairment, conduction disorders, and their associated adverse events related to treatment were defined as main safety specifications. Body weight (BW) and appetite were assessed as effectiveness specifications.

**Results:**

This analysis was based on data as of January 21, 2023. The safety and effectiveness analysis sets included 6016 and 4511 patients, respectively. Treatment‐related adverse events in ≥1% of patients were hyperglycemia (3.9%) and nausea (2.6%). The incidences of hyperglycemia, hepatic impairment, conduction disorders, and their associated adverse events related to treatment were 4.8%, 1.2%, and 1.1%, respectively. The mean changes (standard error [SE]) in BW from baseline to weeks 3, 12, 24, and 52 were 0.64 (0.05) kg, 1.19 (0.12) kg, 1.40 (0.21) kg, and 1.42 (0.39) kg, respectively. The mean changes (SE) in Functional Assessment of Anorexia/Cachexia Treatment 5‐item Anorexia Symptom Scale total scores from baseline to weeks 3, 12, 24, and 52 were 3.2 (0.09), 4.8 (0.18), 5.2 (0.30), and 5.3 (0.47), respectively, exceeding the clinically meaningful improvement score (2.0 points).

**Conclusion:**

The overall safety of anamorelin raised no new safety concerns, although continued caution may be required for hyperglycemia and nausea. Improvements in BW and appetite were also observed in real‐world clinical settings.

## 1 INTRODUCTION

Cancer cachexia is a multifactorial syndrome in patients with cancer characterized by reduced muscle mass and malnutrition that leads to progressive functional impairment^1^ and declined quality of life.^2^ Cancer cachexia also has a significant negative impact on the patient's prognosis.^3^ Clinically, one diagnostic criterion for cancer cachexia includes a reduction in body weight (BW) of ≥5% in the preceding 6 months.^3^

Anamorelin, a highly selective ghrelin receptor agonist,^4^ was approved in Japan in January 2021 for the treatment of cancer cachexia associated with non‐small cell lung cancer (NSCLC), gastric cancer (GC), pancreatic cancer (PC), or colorectal cancer (CRC).^5^ The previous placebo‐controlled study in patients with NSCLC (ONO‐7643‐04)^6^ and the single‐arm study in patients with GC, PC, or CRC (ONO‐7643‐05),^7^ both conducted in Japan, showed that anamorelin improved lean body mass (LBM), BW, and appetite scores determined using the Quality‐of‐Life questionnaire for cancer patients treated with anticancer drugs.^8^ However, those studies included relatively small numbers of patients treated with anamorelin (84 with NSCLC, 40 with CRC, 5 with GC, and 5 with PC) for up to 12 weeks; thus, the safety and effectiveness of treatment with anamorelin beyond 12 weeks remain unclear.^6^, ^7^ Furthermore, there was no established standard for the pharmacological therapy of cancer cachexia.

Therefore, all‐case post‐marketing surveillance (PMS) was conducted in Japan to evaluate the real‐world safety and effectiveness of anamorelin and to help ensure its proper use. In this article, we focused on two clinically important topics: (i) the reproducibility of the results of the clinical trials in the real‐world setting and the absence/presence of new safety concerns, and (ii) the safety and effectiveness after 12 weeks of anamorelin treatment.

Here, we report the results of an interim analysis of the PMS using data collated for 6016 patients who started anamorelin treatment between April 21, 2021 and January 21, 2023. This is the first large‐scale real‐world data of anamorelin in patients with cancer cachexia.

## 2 METHODS

### 2.1 Ethics

This PMS complies with the ministerial ordinance of Good Post‐Marketing Study Practice in Japan. All data were pseudonymized, and all treatment decisions were made by physicians and patients as part of routine clinical practice. According to Japanese guidelines and laws, ethics approval and informed consent were not required for this PMS. Nevertheless, we sought consent from each participating institution to publish the data. This PMS was registered on the National Institute of Public Health/Ministry of Health, Labour and Welfare database (jRCT2011210014; https://rctportal.niph.go.jp/en).

### 2.2 Study design and patients

This ongoing, prospective, non‐interventional, observational study was designed to evaluate the safety and effectiveness of anamorelin in a real‐world setting. All patients who newly started anamorelin since April 21, 2021 were to be registered in this PMS. Patients were to be observed for up to 52 weeks (1 year) after starting anamorelin. In patients who discontinued anamorelin before 52 weeks, the final dosing date was used as the end date of the observation period. We planned to register 5000 patients. For this interim analysis, we included patients who started anamorelin treatment by November 30, 2021, at which time we confirmed that the planned sample size was fulfilled. Patients from facilities that provided consent for publication were included in the safety analysis. Patients with a lack of effectiveness data, and patients with off‐label use of anamorelin, were excluded from the effectiveness analysis.

### 2.3 Assessments

The study assessments included baseline patient characteristics, administration status, safety, and effectiveness. The baseline characteristics included sex, age, BW, body mass index (BMI), Eastern Cooperative Oncology Group Performance Status (ECOG PS), cancer stage, timing of anamorelin administration, performed treatment line, laboratory variables, and comorbidities.

Safety was evaluated in terms of treatment‐related adverse events (TRAEs), which were classified and graded by the attending physicians according to the Japanese version of the Medical Dictionary for Regulatory Activities (version 25.1) and the National Cancer Institute Common Terminology Criteria for Adverse Events (version 5.0). We also assessed the safety specifications (incidences of hyperglycemia, hepatic impairment, conduction disorders, and their associated adverse events related to treatment and interactions with moderate CYP3A4 inhibitors) and the effectiveness specifications (BW and appetite) defined in the risk management plan.

Appetite was evaluated using the Functional Assessment of Anorexia/Cachexia Treatment 5‐item Anorexia Symptom Scale (FAACT‐5IASS),^9^, ^10^ which comprises the following five food‐related questions scored on 5‐point Likert scales:

1. “I have a good appetite”
2. “My interest in food drops as soon as I try to eat”
3. “I have difficulty eating rich or heavy foods”
4. “When I eat, I seem to get full quickly”
5. “Most food tastes unpleasant to me”

The range of each item was from 0 to 4 (0 = not at all; 1 = a little bit; 2 = somewhat; 3 = quite a bit; and 4 = very much). The scores were converted using the formula: [score 1] + (4 − [score 2]) + (4 − [score 3]) + (4 − [score 4]) + (4 − [score 5]), yielding a maximum score of 20. The FACIT and all related works are owned and copyrighted by, and the intellectual property of David Cella, PhD. Permission to use the FAACT questionnaire was obtained by contacting Dr. Cella at information@facit.org. The amount of food intake was also evaluated by a questionnaire, which asked whether food intake was improved, unchanged, or decreased compared to baseline. All data were recorded using case‐report forms (CRFs).

### 2.4 Statistical analyses

All data were analyzed descriptively to determine the number and percentage of patients, or the mean, standard deviation (SD) and standard error (SE), as appropriate. Safety and effectiveness outcomes were analyzed for the overall population and for each indication group separately. If a patient had multiple cancers and the primary cancer could not be determined, the patient was included in the overall population but not in any of the indication groups. Therefore, the sum total of patients included in each indication may not equal the total number of patients in the overall population. All analyses in this surveillance were preplanned. Data were analyzed using SAS version 9.4 (SAS Institute, Cary, NC, USA).

## 3 RESULTS

### 3.1 Patient disposition

**FIGURE 1:**
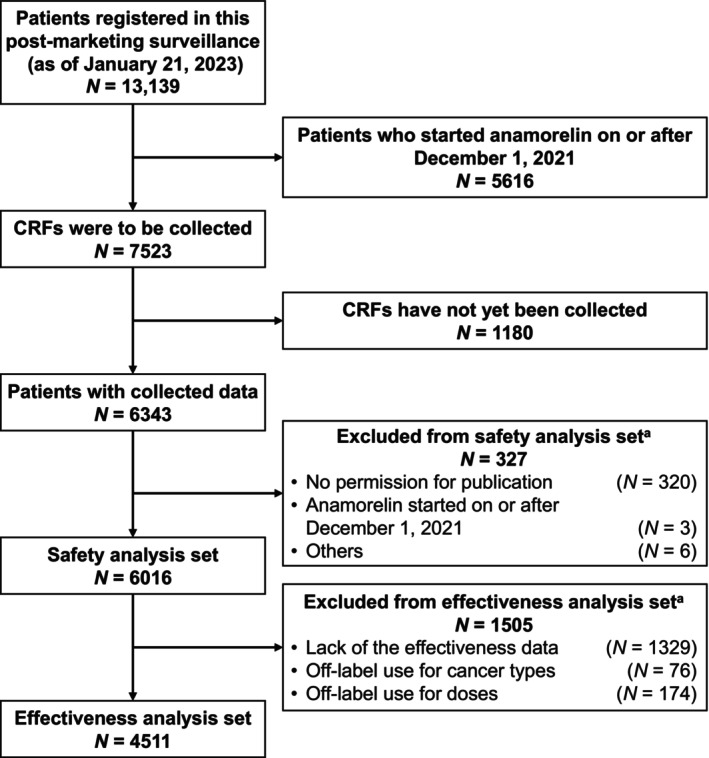
Patient disposition. ^a^Some patients overlapped. CRF, case‐report form.

Between April 21, 2021 and January 21, 2023, a total of 13,139 patients from 1925 institutes had been registered in the PMS (Figure 1). Because registration of the planned sample size (5000 patients) was confirmed to be complete by November 30, 2021, we only included patients who started anamorelin treatment up to December 1, 2021 (*N* = 7523) in this analysis. At this time, the CRFs were fixed for 6343 patients from 1135 institutions. Of those, patients from 1109 institutions that provided consent for publication were included. After excluding patients who were found to have received anamorelin on or after December 1, or those with other reasons, the safety analysis set comprised 6016 patients. The effectiveness analysis set comprised 4511 patients, excluding 1505 patients whose CRFs lacked effectiveness data, or who received off‐label use of anamorelin for other cancer types or at other doses.

### 3.2 Patient characteristics

The characteristics of the 6016 patients in the safety analysis set are presented in Table 1. Among the overall population, 3792 (63.0%) were male. The median age, BW, and BMI were 72 years (range, 25–100 years), 49.2 kg (range, 25.0–97.2 kg), and 19.0 kg/m^2^ (range, 10.9–36.6 kg/m^2^), respectively. A total of 3192 patients (53.1%) had an ECOG PS of 0–1, and 4024 patients (66.9%) received anamorelin alongside anticancer drug therapy. Glucose metabolism disorders, hepatic disorders, and heart disorders were recorded as comorbidities in 17.1%, 3.9%, and 7.0% of patients, respectively. The most common types of comorbidities were diabetes mellitus, hepatic function abnormal, and atrial fibrillation, respectively (Table S1). Regarding cancer type, 1713 patients had NSCLC, 1382 patients had GC, 1617 patients had PC, and 1242 patients had CRC. An ECOG PS of ≥2 was more frequent in patients with NSCLC. A greater proportion of patients with PC were on first‐line therapy compared with patients with other cancers. Glucose metabolism disorders at the start of anamorelin treatment were more frequent in patients with PC.

**TABLE 1:** Patient characteristics according to cancer type.

Characteristics		Overall population^a^ (*N* = 6016)		NSCLC (*N* = 1713)		GC (*N* = 1382)		PC (*N* = 1617)		CRC (*N* = 1242)	
Sex	Male	3792	(63.0)	1172	(68.4)	978	(70.8)	854	(52.8)	744	(59.9)
	Female	2224	(37.0)	541	(31.6)	404	(29.2)	763	(47.2)	498	(40.1)
Age	Median [range]	72 [25–100]		73 [32–100]		72 [25–97]		73 [31–98]		72 [25–100]	
Body weight (kg)	Median [range]	49.2 [25.0–97.2]		49.9 [26.3–97.2]		48.7 [25.1–97.0]		48.3 [25.0–90.6]		50.2 [25.0–94.0]	
BMI (kg/m^2^)	Median [range]	19.0 [10.9–36.6]		19.0 [11.1–35.4]		18.5 [10.9–31.1]		18.9 [11.7–32.1]		19.8 [11.2–36.6]	
ECOG PS	0–1 2 3–4 Unknown	3192 1534 1174 116	(53.1) (25.5) (19.5) (1.9)	728 516 426 43	(42.5) (30.1) (24.9) (2.5)	795 337 230 20	(57.5) (24.4) (16.6) (1.4)	958 352 286 21	(59.2) (21.8) (17.7) (1.3)	679 315 219 29	(54.7) (25.4) (17.6) (2.3)
Stage	I–III IV Recurrent Unknown	577 3947 1423 69	(9.6) (65.6) (23.7) (1.1)	232 1115 351 15	(13.5) (65.1) (20.5) (0.9)	78 905 378 21	(5.6) (65.5) (27.4) (1.5)	212 1107 278 20	(13.1) (68.5) (17.2) (1.2)	49 782 399 12	(3.9) (63.0) (32.1) (1.0)
Treatment period	On drug therapy	4024	(66.9)	1151	(67.2)	993	(71.9)	1053	(65.1)	790	(63.6)
	On BSC	1992	(33.1)	562	(32.8)	389	(28.1)	564	(34.9)	452	(36.4)
Treatment line performed^b^	Yes	4950	(82.3)	1395	(81.4)	1134	(82.1)	1308	(80.9)	1071	(86.2)
	1st line 2nd line 3rd or later line Unknown	1943 1263 1702 42	(39.3) (25.5) (34.4) (0.8)	559 295 532 9	(40.1) (21.1) (38.1) (0.6)	431 272 422 9	(38.0) (24.0) (37.2) (0.8)	641 418 236 13	(49.0) (32.0) (18.0) (1.0)	291 269 502 9	(27.2) (25.1) (46.9) (0.8)
Comorbidities	Glucose metabolism disorders	1029	(17.1)	250	(14.6)	161	(11.6)	451	(27.9)	152	(12.2)
	Hepatic disorders	232	(3.9)	77	(4.5)	41	(3.0)	76	(4.7)	37	(3.0)
	Heart disorders	420	(7.0)	153	(8.9)	87	(6.3)	107	(6.6)	70	(5.6)

*Note*: Values are *n* (%) of patients, unless otherwise specified.

Abbreviations: BMI, body mass index; BSC, best supportive care; CRC, colorectal cancer; CRP, C‐reactive protein; ECOG PS, Eastern Cooperative Oncology Group Performance Status; GC, gastric cancer; NSCLC, non‐small cell lung cancer; PC, pancreatic cancer.

^a^ The overall population includes patients with cancers other than NSCLC, GC, PC, and CRC.

^b^ This population includes patients on BSC.

### 3.3 Duration of anamorelin treatment and reasons for discontinuation

The duration of anamorelin treatment in the overall population was 0–3 weeks in 41.9% of patients, 4–12 weeks in 39.7%, 13–24 weeks in 9.6%, and 25–52 weeks in 8.8% (Table 2). The median treatment duration of anamorelin was 29 days overall, and was similar among patients with NSCLC, GC, PC, and CRC (28–33 days). The reasons for discontinuation of anamorelin are listed in Table S2. In the overall population, the most common reason for discontinuation within 0–52 weeks was cancer progression (excluding death) in 30.8% of patients, followed by poor response to anamorelin treatment in 22.1%, and adverse events in 15.9%, while 9.6% of patients discontinued because they had an effective response to anamorelin treatment. According to the reasons for discontinuation by timepoint, the percentage of patients who discontinued due to adverse events was greatest in weeks 0–3, but it declined over time. By comparison, the percentage of patients who discontinued anamorelin due to an effective response tended to be greater in weeks 4–12 and 13–52 than in weeks 0–3.

**TABLE 2:** Duration of anamorelin treatment according to cancer type.

Duration of treatment	Overall population^a^ (*N* = 6015)		NSCLC (*N* = 1712)		GC (*N* = 1382)		PC (*N* = 1617)		CRC (*N* = 1242)	
0–3 weeks	2519	(41.9)	668	(39.0)	581	(42.0)	732	(45.3)	503	(40.5)
4–12 weeks	2385	(39.7)	734	(42.9)	529	(38.3)	595	(36.8)	510	(41.0)
13–24 weeks	579	(9.6)	163	(9.5)	143	(10.3)	150	(9.3)	118	(9.5)
25–52 weeks	532	(8.8)	147	(8.6)	129	(9.3)	140	(8.7)	111	(8.9)
Median [range] days	29 [1–365]		33 [1–365]		29 [1–365]		28 [1–365]		29 [1–365]	

*Note*: Values are *n* (%) of patients, unless otherwise specified.

Abbreviations: CRC, colorectal cancer; GC, gastric cancer; NSCLC, non‐small cell lung cancer; PC, pancreatic cancer.

^a^ The overall population includes patients with cancers other than NSCLC, GC, PC and CRC.

### 3.4 Safety

TRAEs occurred in 884 patients (14.7%) and grade ≥3 TRAEs occurred in 203 patients (3.4%) in the overall population (Table 3). TRAEs in ≥1% of patients were hyperglycemia (any grade: 3.9%; grade ≥3: 1.6%) and nausea (any grade: 2.6%; grade ≥3: 0.3%). Patients with PC had a higher frequency of hyperglycemia (any grade: 6.5%; grade ≥3: 3.4%) than patients with other cancers. Grade 5 TRAEs were reported in two patients with GC (pneumonia, arrhythmia), one patient with PC (pulmonary edema), and one patient with CRC (pneumonia aspiration).

**TABLE 3:** Anamorelin treatment‐related adverse events according to cancer type.

Safety specifications	Overall population^a^ (*N* = 6016)				NSCLC (*N* = 1713)				GC (*N* = 1382)				PC (*N* = 1617)				CRC (*N* = 1242)			
	Any grade		Grade ≥3^b^		Any grade		Grade ≥3^b^		Any grade		Grade ≥3^b^		Any grade		Grade ≥3^b^		Any grade		Grade ≥3^b^	
Any TRAE	884	(14.7)	203	(3.4)	269	(15.7)	52	(3.0)	164	(11.9)	34	(2.5)	275	(17.0)	77	(4.8)	170	(13.7)	38	(3.1)
TRAEs (in ≥1% of patients)																				
Hyperglycemia	234	(3.9)	99	(1.6)	60	(3.5)	20	(1.2)	36	(2.6)	12	(0.9)	105	(6.5)	55	(3.4)	31	(2.5)	11	(0.9)
Nausea Safety specifications	156	(2.6)	16	(0.3)	37	(2.2)	3	(0.2)	32	(2.3)	5	(0.4)	53	(3.3)	3	(0.2)	33	(2.7)	5	(0.4)
Hyperglycemia‐associated TRAEs	288	(4.8)	118	(2.0)	82	(4.8)	26	(1.5)	45	(3.3)	16	(1.2)	118	(7.3)	61	(3.8)	39	(3.1)	14	(1.1)
Hepatic disorders‐associated TRAEs	70	(1.2)	16	(0.3)	28	(1.6)	7	(0.4)	15	(1.1)	2	(0.1)	13	(0.8)	1	(0.1)	14	(1.1)	6	(0.5)
Conduction disorders‐associated TRAEs	65	(1.1)	18	(0.3)	28	(1.6)	7	(0.4)	14	(1.0)	3	(0.2)	10	(0.6)	3	(0.2)	13	(1.0)	5	(0.4)

*Note*: Values are *n* (%) of patients.

Abbreviations: CRC, colorectal cancer; GC, gastric cancer; NSCLC, non‐small cell lung cancer; PC, pancreatic cancer; TRAE, treatment‐related adverse event.

^a^ The overall population includes patients with cancers other than NSCLC, GC, PC, and CRC.

^b^ Grade 5 TRAEs were reported in two patients with GC (pneumonia, arrhythmia), one patient with PC (pulmonary edema), and one patient with CRC (pneumonia aspiration).

Regarding the safety specifications, the frequencies of hyperglycemia, hepatic impairment, conduction disorders, and their associated adverse events related to treatment were 4.8% (grade ≥3: 2.0%), 1.2% (grade ≥3: 0.3%), and 1.1% (grade ≥3: 0.3%), respectively. The most common hyperglycemia, hepatic impairment, conduction disorders, and their associated adverse events related to treatment were hyperglycemia, alanine aminotransferase increased, and electrocardiogram QT prolonged, respectively (Table S3). The frequencies of hyperglycemia, hepatic impairment, conduction disorders, and their associated adverse events related to treatment were higher in patients with concomitant glucose metabolism disorders, hepatic disorders, or heart disorders, respectively, than in patients without these disorders (Table S4). Additionally, most of the TRAEs included in the safety specifications occurred within 16 weeks from the start of anamorelin treatment (Table S5).

### 3.5 Effectiveness

#### 3.5.1 Body weight

**FIGURE 2:**
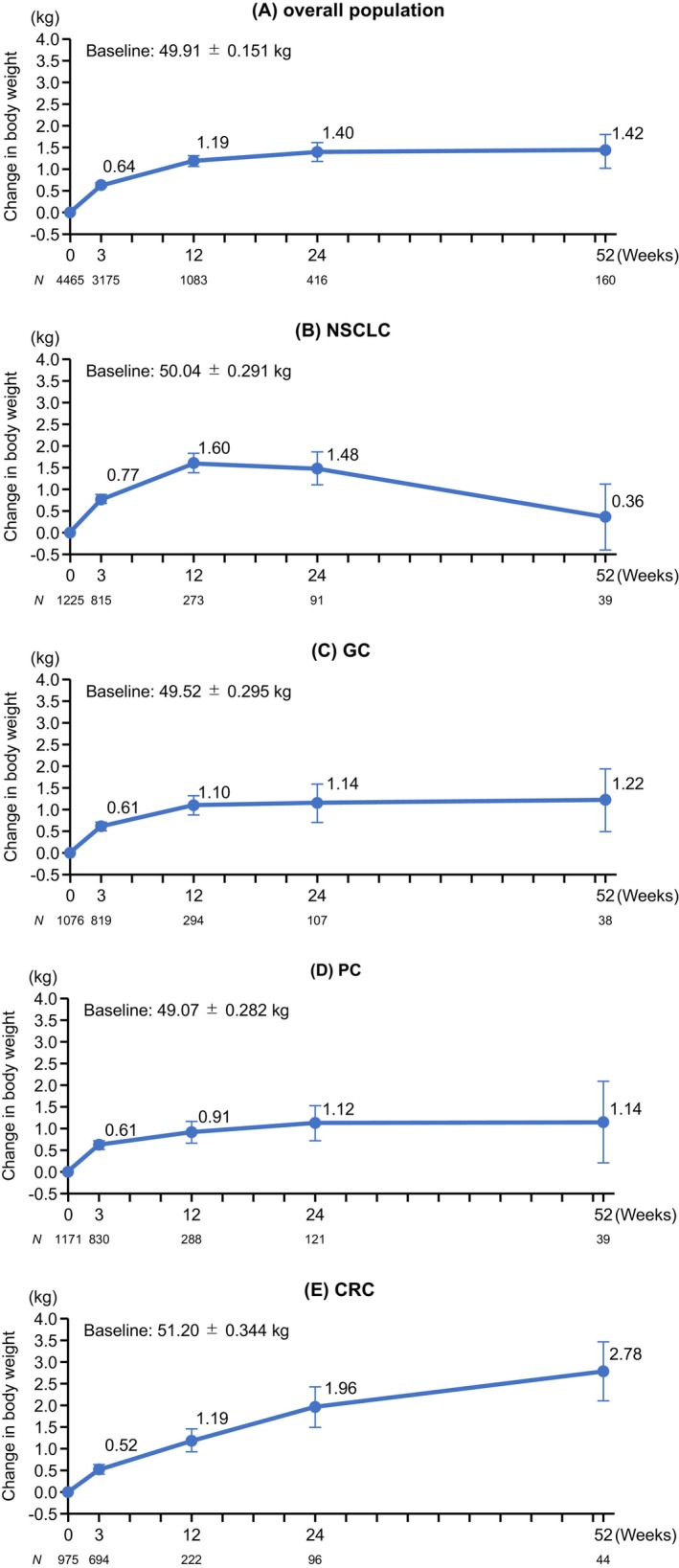
Changes in body weight from baseline in the overall population (A), and in patients with NSCLC (B), GC (C), PC (D), or CRC (E). Values are means ± standard error. CRC, colorectal cancer; GC, gastric cancer; NSCLC, non‐small cell lung cancer; PC, pancreatic cancer.

In the overall population, the mean changes (SE) in BW from baseline to weeks 3, 12, 24, and 52 were 0.64 (0.05) kg, 1.19 (0.12) kg, 1.40 (0.21) kg, and 1.42 (0.39) kg, respectively (Figure 2). The mean changes (SE) from baseline to week 12 in patients with NSCLC, GC, PC, and CRC were 1.60 (0.22) kg, 1.10 (0.23) kg, 0.91 (0.25) kg, and 1.19 (0.26) kg, respectively. The mean changes (SE) from baseline to week 52 in patients with NSCLC, GC, PC, and CRC were 0.36 (0.76) kg, 1.22 (0.73) kg, 1.14 (0.94) kg, and 2.78 (0.68) kg, respectively.

The mean rates of change in BW according to patient background characteristics are summarized in Table S6. In the overall population, the mean rates of change from baseline to week 12 were increased in almost all subgroups of patients (age <75 or ≥75; ECOG PS of 0–1, 2, or 3–4; BMI of <20 kg/m^2^ or ≥20 kg/m^2^; on anticancer drug therapy or on best supportive care; with or without immune checkpoint inhibitors; with or without steroids; without history of gastric surgery, with total gastrectomy, or with partial gastrectomy). However, the mean rate of change in BW from baseline to week 12 was not increased in the ≥20 kg/m^2^ subgroup (0.10% decrease).

#### 3.5.2 Appetite

**FIGURE 3:**
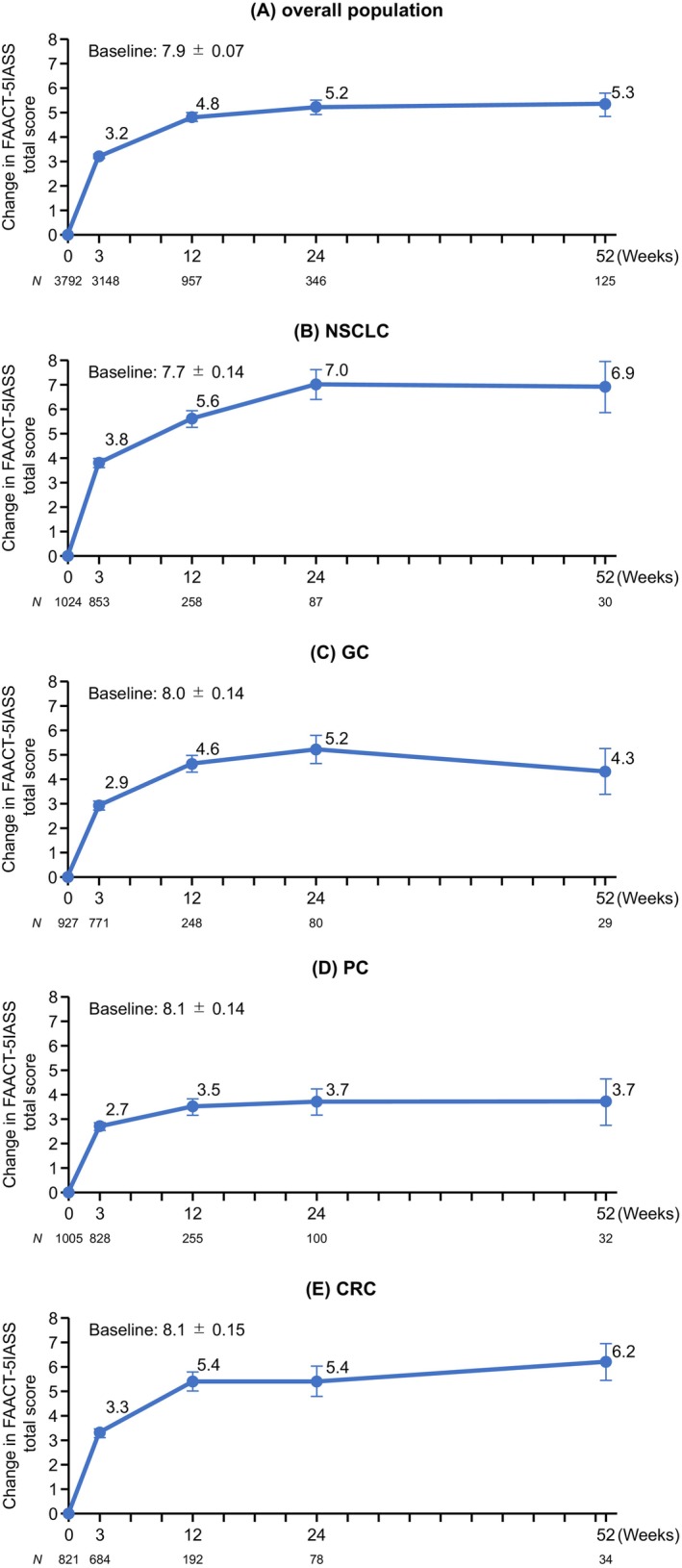
Changes in FAACT‐5IASS total scores from baseline in the overall population (A), and in patients with NSCLC (B), GC (C), PC (D), or CRC (E). Values are means ± standard error. CRC, colorectal cancer; FAACT‐5IASS, Functional Assessment of Anorexia/Cachexia Therapy 5‐item Anorexia Symptom Scale; GC, gastric cancer; NSCLC, non‐small cell lung cancer; PC, pancreatic cancer.

Figure 3 shows the changes in the FAACT‐5IASS total score from baseline. It was previously reported that an increase in the total score by ≥2 points from baseline represents a clinically meaningful improvement.^11^ In the overall population, a clinically meaningful improvement in the FAACT‐5IASS total score was observed at each timepoint, with mean changes (SE) from baseline to weeks 3, 12, 24, and 52 of 3.2 (0.09), 4.8 (0.18), 5.2 (0.30), and 5.3 (0.47), respectively. The mean changes (SE) from baseline to week 12 in patients with NSCLC, GC, PC, and CRC were 5.6 (0.34), 4.6 (0.35), 3.5 (0.34), and 5.4 (0.38), respectively. The mean changes (SE) from baseline to week 52 in patients with NSCLC, GC, PC, and CRC were 6.9 (1.04), 4.3 (0.93), 3.7 (0.96), and 6.2 (0.74), respectively. In subgroup analyses by patient background characteristics, the mean changes in the FAACT‐5IASS total score from baseline to week 12 increased in all subgroups (Table S7).

Regarding food intake, the percentage of patients who felt that their food intake had increased at week 12 relative to baseline was 62.2% in the overall population (Figure S1), 67.2% of patients with NSCLC, 61.5% of patients with GC, 56.9% of patients with PC, and 63.0% of patients with CRC.

## 4 DISCUSSION

These interim results of the PMS represent the largest‐scale real‐world data for anamorelin treatment in patients with cancer cachexia associated with NSCLC, GC, PC, or CRC. These results were based on the safety data from over 6000 patients and the effectiveness data were based on data from over 4500 patients. Although some studies have examined the real‐world safety and effectiveness of anamorelin, those studies included small numbers of patients with GC, PC, or CRC.^12^, ^13^, ^14^ Thus, our results help to fill the data gap between the previous clinical trials and the real‐world clinical outcomes, providing comprehensive insights into the safety and effectiveness of anamorelin that will be valuable for future clinical practice.

The treatment lines in which anamorelin was used varied among the four cancer types (Table 1). The use of anamorelin alongside first‐line anticancer treatment was more common in patients with PC (49.0%) than in patients with NSCLC (40.1%), GC (38.0%), or CRC (27.2%). One possible reason for this may be the difference in the timing of onset of cancer cachexia. A previous prospective observational study in Japan reported that 31.7% of Japanese patients with previously untreated advanced NSCLC had cachexia.^15^ However, a previous single‐center, retrospective, observational study of Japanese patients with PC reported that the incidence of cachexia at the start of first‐line chemotherapy was 50%,^16^ which was higher than that in patients with NSCLC.^15^

This PMS included a larger proportion of patients with an ECOG PS of 2 (22.5%) or 3–4 (19.5%) than the previous clinical trials in Japan (ONO‐7643‐04 and ONO‐7643‐05), in which 12% and 10.2% of patients had an ECOG PS of 2, respectively.^6^, ^7^ In addition, patients with PC who had an ECOG PS of 2–4, and patients with NSCLC, GC, and CRC who had an ECOG PS of 3–4 were excluded from the ONO‐7643‐04 and ONO‐7643‐05 trials. Moreover, the follow‐up periods in those clinical trials were limited to ≤16 weeks after anamorelin administration. Despite the greater number of patients with ECOG PS of ≥2 and the longer follow‐up period in the current analysis, the overall frequency of TRAEs in this PMS (14.7%; grade ≥3: 3.4%) was not numerically greater than the corresponding results in the previous clinical trials (adverse drug reactions in ONO‐7643‐04: 41.0% [grade ≥3, 7.2%]; TRAEs in ONO‐7643‐05: 42.9% [grade ≥3, 10.2%]).^6^, ^7^

Regarding the safety specifications of anamorelin, we observed a trend that patients with particular comorbidities had a higher incidence of hyperglycemia, hepatic impairment, conduction disorders, and their associated adverse events related to treatment. In addition, we observed that the patients with PC tended to have a higher incidence of hyperglycemia and its associated adverse events related to treatment (7.6%) than those with other cancer types, which was consistent with the results of a previous retrospective observational study.^12^ A study using a Japanese administrative claims database of patients with PC who were treated with anamorelin showed that history of diabetes mellitus was associated with increased adverse glucose metabolic effects.^17^ In the current study, the rate of glucose metabolism disorders at baseline (27.9%) was higher in patients with PC than in those with other cancers, which potentially contributed to the higher incidence of hyperglycemia and associated TRAEs. Taken together, while the overall safety results raised no new safety concerns, careful monitoring and management may be required for hyperglycemia and its associated events, especially in patients with PC. We also need to mention that the only TRAE that occurred in >1% of patients, other than the safety specifications, was nausea (2.6% of the overall population). Since the majority of the patients in the current study received anamorelin while on other anticancer treatments, it is possible that the concomitant anticancer drugs may have influenced the development of nausea. However, because we cannot deny an association between the occurrence of nausea and anamorelin treatment, further evaluation may be required to clarify the mechanism involved in the development of nausea.

Regarding the effectiveness of anamorelin, we observed an improvement (gain) in BW in the overall population from baseline to week 3 (0.64 kg) and week 12 (1.19 kg), confirming the previous results of Japanese clinical trials.^6^, ^7^ Furthermore, the effect of anamorelin on BW was well maintained in the overall population beyond 12 weeks, because the increase in BW was apparent at week 24 (1.60 kg) and at week 52 (1.42 kg). Among patients with NSCLC, the mean change in BW from baseline to week 12 was 1.60 kg; however, the magnitude of the BW gain gradually became smaller until week 52 (0.36 kg). Of note, the percentage of patients with NSCLC who discontinued anamorelin due to an effective response at each timepoint was higher than for patients with other cancers (Table S2). Therefore, the smaller BW gain between weeks 13 and 52 may be attributed to the discontinuation of patients who experienced a clinically relevant BW gain. Nevertheless, while a difference in BW responses among cancer types was apparent at the later timepoints, the effectiveness of anamorelin was generally observed across all cancer types. The differences in BW responses among the cancer types may be associated with the progress of the cancer itself, which is affected by the effectiveness of anticancer treatment for those who are on drug therapy. In particular, CRC is often treatable with various therapeutic options, whereas the other cancer types are harder to treat. Therefore, the relatively better BW response in patients with CRC may be associated with the effect of anticancer treatment. However, because we did not collect information regarding the effectiveness of anticancer treatment in this PMS, future studies may dissect how the progression or treatment of cancer affects the BW response to anamorelin. The impact of anamorelin on the eventual treatment outcomes of concomitant anticancer treatment may be even more important for patients with cancer. Although this PMS did not evaluate survival outcomes, several observational studies are underway to investigate the impact of anamorelin on the effectiveness of anticancer treatment for NSCLC or gastrointestinal cancer.^18^, ^19^, ^20^ Results from those studies will be useful for guiding clinical decisions in the future.

Regarding appetite, the mean changes in FAACT‐5IASS total scores from baseline to weeks 3 and 12 showed improvements in the overall population (3.2 at week 3 and 4.8 at week 12). Further, the improvement in FAACT‐5IASS total scores was maintained beyond 12 weeks after the start of anamorelin treatment, with a mean change of 5.2 at week 24 and 5.3 at week 52. Notably, the score increased by ≥2 points, which was described as a clinically meaningful improvement,^11^ at all timepoints in all subgroups of patients. A previous Japanese clinical trial evaluated the effectiveness of anamorelin using FAACT‐5IASS in patients with cancer cachexia and low BMI.^21^ The FAACT‐5IASS score increased at week 3 and the increase was sustained through to week 24, in agreement with our results. Furthermore, >50% of patients in the overall population reported that their food intake had increased at weeks 3, 12, 24, and 52 relative to baseline. Although the effectiveness of anamorelin was primarily assessed in terms of the improvements in LBM, BW, and appetite in prior studies, the amount of food intake may also be helpful for evaluating the effectiveness of anamorelin in the future.

According to the subgroup effectiveness analysis by patient background characteristics, most of the subgroups showed tendencies towards increases in BW and appetite from baseline (Table S6,S7), although patients with a BMI <20 kg/m^2^ showed a greater BW gain than patients with a BMI ≥20 kg/m^2^. Post hoc subgroup analyses of the ONO‐7643‐04 trial revealed that the odds ratio for BW improvement in the anamorelin arm vs. placebo arm tended to be greater in patients with a BMI of <20 kg/m^2^ at baseline than in those with a BMI of ≥20 kg/m^2^,^22^ consistent with the current results. Although an increased appetite was observed regardless of baseline BMI, a baseline BMI of <20 kg/m^2^ may be useful as a predictor of BW gain in patients treated with anamorelin. Future studies examining the predictors for the effectiveness of treatment may help clinical decisions regarding the use of anamorelin.

## 5 LIMITATIONS

This PMS study has several limitations. First, all information about TRAEs was reported by the physicians, and was not verified in terms of their grade or seriousness by central review. Second, the effectiveness data were only analyzed for patients who had relevant data and could continue anamorelin treatment at each timepoint, potentially causing selection bias. Finally, since this is an interim analysis, the results of the final analysis may differ from the current results.

## 6 CONCLUSIONS

Based on the results of this interim analysis, the overall safety of anamorelin raised no new safety concerns, but continued caution may be required for hyperglycemia and nausea. Improvements in BW and appetite were also observed in real‐world clinical settings and, in particular, these improvements persisted in patients who continued anamorelin treatment for more than 12 weeks.

## AUTHOR CONTRIBUTIONS

**Koichi Takayama:** Conceptualization (supporting); writing – review and editing (supporting). **Ai Kojima:** Conceptualization (supporting); data curation (equal); investigation (lead); methodology (equal); project administration (lead); writing – review and editing (supporting). **Chikara Honda:** Conceptualization (supporting); data curation (equal); formal analysis (equal); investigation (supporting); methodology (equal); project administration (supporting); resources (equal); writing – original draft (supporting); writing – review and editing (supporting). **Masahiro Nakayama:** Conceptualization (lead); data curation (equal); investigation (lead); methodology (equal); project administration (lead); writing – review and editing (supporting). **Satomi Kanemata:** Visualization (supporting); writing – original draft (supporting); writing – review and editing (lead). **Toshimitsu Endo:** Visualization (lead); writing – original draft (lead); writing – review and editing (lead). **Kei Muro:** Conceptualization (supporting); writing – review and editing (supporting).

## FUNDING INFORMATION

This study was funded by Ono Pharmaceutical Co., Ltd.

## CONFLICT OF INTEREST STATEMENT

Koichi Takayama reports a research grant to the institution for this PMS from Ono Pharmaceutical; research grants to the institution from Eli Lilly and Taiho Pharmaceutical; lecture fees from AstraZeneca, Boehringer‐Ingelheim, Chugai‐Roche, Daiichi Sankyo, Eli Lilly, MSD‐Merck, and Ono Pharmaceutical; and is the Director of the Japan Lung Cancer Society. Kei Muro reports a research grant to the institution for this PMS from Ono Pharmaceutical; research grants to the institution from Amgen, Ono Pharmaceutical, Astellas, Sanofi, Taiho, PRA Health Sciences, PAREXEL International, Novartis, Chugai, and MSD; consulting fees from Amgen, AstraZeneca, Ono Pharmaceutical, Astellas, and Chugai; lecture fees from Ono Pharmaceutical, Taiho, Bristol‐Myers Squibb, Eli Lilly, MSD, Takeda, and Daiichi Sankyo; and is a member of advisory boards for Astellas, Amgen, AstraZeneca, Eli Lilly, and Takeda. Ai Kojima, Chikara Honda, Masahiro Nakayama, Satomi Kanemata, and Toshimitsu Endo are employees of Ono Pharmaceutical.

## ETHICS STATEMENT

This PMS was designed and conducted in accordance with Japanese Guidelines for Post‐Marketing Surveillance. Under Japanese guidelines and laws, ethics approval was not required for the purpose of this PMS.

## CONSENT

This PMS was designed and conducted in accordance with Japanese Guidelines for Post‐Marketing Surveillance. Under Japanese guidelines and laws, informed consent was not required for the purpose of this PMS. Consent for using patient data was obtained from the participating sites.

## Supporting information


Data S1.

## Data Availability

The data sets analyzed in this article are not publicly available because patient consent for individual data disclosure has not been obtained. However, data are available from the corresponding author on reasonable request.
