# Supplementary material for: Real‐world safety and effectiveness of anamorelin for cancer cachexia: Interim analysis of post‐marketing surveillance in Japan
Source: Cancer Med. 2024 May 1;13(9):e7170. doi: 10.1002/cam4.7170 (PMC11063721; doi:10.1002/cam4.7170)
Supplement: Supplementary file 1 — Data S1. [file CAM4-13-e7170-s001.docx]

# Supporting Information

**Contents**

|  |
| --- |
| **Table S1.** Details of the types of glucose metabolism disorders, hepatic disorders, and heart disorders reported as comorbidities in individual patients |
| **Table S2.** Reasons for discontinuation of anamorelin according to cancer type and timepoint |
| **Table S3.** Incidence of treatment-related adverse events listed in the safety specifications |
| **Table S4.** Incidence of treatment-related adverse events listed in the safety specifications among patients with the indicated comorbidities |
| **Table S5.** Incidence rate of the safety specifications by timepoint |
| **Table S6.** Percent changes in body weight according to patient background characteristics |
| **Table S7.** Changes in FAACT-5IASS total scores according to patient background characteristics |
| **Figure S1.** Changes in food intake relative to the baseline in the overall population (A), and in patients with NSCLC (B), GC (C), PC (D), or CRC (E) |

**Table S1.** Details of the types of glucose metabolism disorders, hepatic disorders, and heart disorders reported as comorbidities in individual patients

| **System organ class** | ***N*** | **Preferred terms** | ***N*** |
| --- | --- | --- | --- |
| **Glucose metabolism disorders** | 1031 |  |  |
| Metabolism and nutrition disorders | 1031 | Diabetes mellitus | 762 |
|  |  | Glucose tolerance impaired | 10 |
|  |  | Hyperglycemia | 6 |
|  |  | Hypoglycemia | 1 |
|  |  | Pancreatogenous diabetes | 15 |
|  |  | Type 1 diabetes mellitus | 5 |
|  |  | Type 2 diabetes mellitus | 228 |
|  |  | Fulminant type 1 diabetes mellitus | 1 |
|  |  | Steroid diabetes | 3 |
| **Hepatic disorders** | 252 |  |  |
| Infections and infestations | 64 | Chronic hepatitis B | 10 |
|  |  | Chronic hepatitis C | 5 |
|  |  | Hepatitis A | 0 |
|  |  | Hepatitis B | 24 |
|  |  | Hepatitis C | 6 |
|  |  | Hepatitis viral | 1 |
|  |  | Liver abscess | 3 |
|  |  | Viral hepatitis carrier | 14 |
|  |  | Hepatitis B reactivation | 1 |
|  |  | Acute hepatitis B | 0 |
| Neoplasms benign, malignant and unspecified (incl cysts and polyps) | 4 | Hemangioma of liver | 3 |
|  |  | Hepatic neoplasm | 1 |
| Metabolism and nutrition disorders | 1 | Hyperammonemia | 1 |
| Nervous system disorders | 1 | Hepatic encephalopathy | 1 |
| Gastrointestinal disorders | 24 | Ascites | 21 |
|  |  | Esophageal varices hemorrhage | 0 |
|  |  | Gastric varices | 0 |
|  |  | Varices esophageal | 3 |
| Hepatobiliary disorders | 157 | Alcoholic liver disease | 15 |
|  |  | Autoimmune hepatitis | 3 |
|  |  | Cholestasis | 3 |
|  |  | Chronic hepatitis | 1 |
|  |  | Cirrhosis alcoholic | 4 |
|  |  | Fatty liver alcoholic | 0 |
|  |  | Hepatic cirrhosis | 7 |
|  |  | Hepatic cyst | 2 |
|  |  | Hepatic failure | 1 |
|  |  | Hepatic function abnormal | 52 |
|  |  | Hepatic steatosis | 18 |
|  |  | Hepatitis | 2 |
|  |  | Hepatitis acute | 0 |
|  |  | Hepatitis alcoholic | 1 |
|  |  | Hyperbilirubinemia | 1 |
|  |  | Jaundice | 1 |
|  |  | Jaundice cholestatic | 14 |
|  |  | Liver disorder | 17 |
|  |  | Non-alcoholic fatty liver | 1 |
|  |  | Drug-induced liver injury | 8 |
|  |  | Primary biliary cholangitis | 6 |
| General disorders and administration site conditions | 1 | Edema due to hepatic disease | 1 |
| **Heart disorders** | 485 |  |  |
| Infections and infestations | 2 | Endocarditis | 2 |
| Neoplasms benign, malignant and unspecified (incl cysts and polyps) | 3 | Pericarditis malignant | 2 |
|  |  | Cardiac myxoma | 1 |
| Cardiac disorders | 419 | Acute myocardial infarction | 0 |
|  |  | Angina pectoris | 55 |
|  |  | Angina unstable | 3 |
|  |  | Aortic valve incompetence | 4 |
|  |  | Aortic valve stenosis | 6 |
|  |  | Arrhythmia | 16 |
|  |  | Arteriosclerosis coronary artery | 5 |
|  |  | Atrial fibrillation | 141 |
|  |  | Atrial flutter | 4 |
|  |  | Atrioventricular block | 2 |
|  |  | Atrioventricular block complete | 2 |
|  |  | Atrioventricular block first degree | 6 |
|  |  | Atrioventricular block second degree | 1 |
|  |  | Bundle branch block left | 1 |
|  |  | Bundle branch block right | 4 |
|  |  | Cardiac amyloidosis | 1 |
|  |  | Cardiac aneurysm | 1 |
|  |  | Cardiac failure | 17 |
|  |  | Cardiac failure chronic | 31 |
|  |  | Cardiac failure congestive | 9 |
|  |  | Cardiac hypertrophy | 1 |
|  |  | Cardiac tamponade | 1 |
|  |  | Cardiomegaly | 1 |
|  |  | Cardiomyopathy alcoholic | 1 |
|  |  | Coronary artery disease | 2 |
|  |  | Coronary artery stenosis | 3 |
|  |  | Extrasystoles | 1 |
|  |  | Mitral valve incompetence | 7 |
|  |  | Mitral valve prolapse | 0 |
|  |  | Mitral valve stenosis | 0 |
|  |  | Myocardial infarction | 26 |
|  |  | Myocardial ischemia | 7 |
|  |  | Myocarditis | 0 |
|  |  | Prinzmetal angina | 8 |
|  |  | Sinus bradycardia | 1 |
|  |  | Sinus tachycardia | 3 |
|  |  | Supraventricular extrasystoles | 4 |
|  |  | Supraventricular tachycardia | 7 |
|  |  | Tachycardia | 1 |
|  |  | Tachycardia paroxysmal | 1 |
|  |  | Tricuspid valve incompetence | 2 |
|  |  | Ventricular extrasystoles | 3 |
|  |  | Ventricular tachycardia | 2 |
|  |  | Wolff-Parkinson-White syndrome | 1 |
|  |  | Tachyarrhythmia | 5 |
|  |  | Left ventricular dysfunction | 1 |
|  |  | Bradyarrhythmia | 0 |
|  |  | Acute coronary syndrome | 1 |
|  |  | Cardiac ventricular thrombosis | 1 |
|  |  | Congestive cardiomyopathy | 6 |
|  |  | Cardiac valve disease | 3 |
|  |  | Aortic valve disease | 1 |
|  |  | Stress cardiomyopathy | 3 |
|  |  | Sinus node dysfunction | 6 |
|  |  | Immune-mediated myocarditis | 0 |
| Gastrointestinal disorders | 21 | Ascites | 21 |
| Congenital, familial and genetic disorders | 11 | Atrial septal defect | 1 |
|  |  | Hypertrophic cardiomyopathy | 9 |
|  |  | Ventricular septal defect | 0 |
|  |  | Aortic valve atresia | 1 |
| General disorders and administration site conditions | 28 | Edema peripheral | 28 |
| Investigations | 1 | Blood pressure abnormal | 0 |
|  |  | Blood pressure decreased | 1 |

*Note:* Values are the numbers of comorbidities.

**Table S2.** Reasons for discontinuation of anamorelin according to cancer type and timepoint

|  |  | **0–3 weeks** | | **4–12 weeks** | | **13–52 weeks** | | **0–52 weeks** | |
| --- | --- | --- | --- | --- | --- | --- | --- | --- | --- |
| **Overall population ^a^** |  |  |  |  |  |  |  |  |  |
| Total | *N* | 6016 | | 3497 | | 1113 | | 6016 | |
| Patients who discontinued |  | 2519 | (41.9) | 2384 | (68.2) | 927 | (83.3) | 5830 | (96.9) |
| Reasons for discontinuation ^b^ | Cancer progression (excluding death) | 741 | (12.3) | 752 | (21.5) | 360 | (32.3) | 1853 | (30.8) |
|  | Poor response | 658 | (10.9) | 536 | (15.3) | 136 | (12.2) | 1330 | (22.1) |
|  | Adverse events | 620 | (10.3) | 285 | (8.1) | 50 | (4.5) | 955 | (15.9) |
|  | Death | 271 | (4.5) | 297 | (8.5) | 128 | (11.5) | 696 | (11.6) |
|  | Department/hospital transfer | 219 | (3.6) | 276 | (7.9) | 100 | (9.0) | 595 | (9.9) |
|  | Effective response | 117 | (1.9) | 288 | (8.2) | 173 | (15.5) | 578 | (9.6) |
|  | Lost to follow-up | 17 | (0.3) | 36 | (1.0) | 12 | (1.1) | 65 | (1.1) |
|  | Other ^c^ | 358 | (6.0) | 278 | (7.9) | 68 | (6.1) | 704 | (11.7) |
| **NSCLC** |  |  |  |  |  |  |  |  |  |
| Total | *N* | 1713 | | 1045 | | 311 | | 1713 | |
| Patients who discontinued |  | 668 | (39.0) | 734 | (70.2) | 258 | (83.0) | 1660 | (96.9) |
| Reasons for discontinuation ^b^ | Cancer progression (excluding death) | 165 | (9.6) | 191 | (18.3) | 79 | (25.4) | 435 | (25.4) |
|  | Poor response | 191 | (11.2) | 164 | (15.7) | 43 | (13.8) | 398 | (23.2) |
|  | Adverse events | 176 | (10.3) | 84 | (8.0) | 14 | (4.5) | 274 | (16.0) |
|  | Death | 69 | (4.0) | 86 | (8.2) | 41 | (13.2) | 196 | (11.4) |
|  | Department/hospital transfer | 53 | (3.1) | 99 | (9.5) | 23 | (7.4) | 175 | (10.2) |
|  | Effective response | 45 | (2.6) | 121 | (11.6) | 63 | (20.3) | 229 | (13.4) |
|  | Lost to follow-up | 3 | (0.2) | 15 | (1.4) | 2 | (0.6) | 20 | (1.2) |
|  | Other ^c^ | 95 | (5.5) | 81 | (7.8) | 19 | (6.1) | 195 | (11.4) |
| **GC** |  |  |  |  |  |  |  |  |  |
| Total | *N* | 1382 | | 801 | | 273 | | 1382 | |
| Patients who discontinued |  | 581 | (42.0) | 528 | (65.9) | 230 | (84.2) | 1339 | (96.9) |
| Reasons for discontinuation ^b^ | Cancer progression (excluding death) | 193 | (14.0) | 189 | (23.6) | 95 | (34.8) | 477 | (34.5) |
|  | Poor response | 134 | (9.7) | 126 | (15.7) | 36 | (13.2) | 296 | (21.4) |
|  | Adverse events | 128 | (9.3) | 52 | (6.5) | 11 | (4.0) | 191 | (13.8) |
|  | Death | 81 | (5.9) | 70 | (8.7) | 29 | (10.6) | 180 | (13.0) |
|  | Department/hospital transfer | 46 | (3.3) | 49 | (6.1) | 22 | (8.1) | 117 | (8.5) |
|  | Effective response | 19 | (1.4) | 49 | (6.1) | 37 | (13.6) | 105 | (7.6) |
|  | Lost to follow-up | 6 | (0.4) | 10 | (1.2) | 4 | (1.5) | 20 | (1.4) |
|  | Other ^c^ | 80 | (5.8) | 64 | (8.0) | 19 | (7.0) | 163 | (11.8) |
| **PC** |  |  |  |  |  |  |  |  |  |
| Total | *N* | 1617 | | 885 | | 290 | | 1617 | |
| Patients who discontinued |  | 732 | (45.3) | 595 | (67.2) | 246 | (84.8) | 1573 | (97.3) |
| Reasons for discontinuation ^b^ | Cancer progression (excluding death) | 219 | (13.5) | 202 | (22.8) | 98 | (33.8) | 519 | (32.1) |
|  | Poor response | 191 | (11.8) | 122 | (13.8) | 32 | (11.0) | 345 | (21.3) |
|  | Adverse events | 208 | (12.9) | 75 | (8.5) | 12 | (4.1) | 295 | (18.2) |
|  | Death | 68 | (4.2) | 78 | (8.8) | 43 | (14.8) | 189 | (11.7) |
|  | Department/hospital transfer | 76 | (4.7) | 77 | (8.7) | 32 | (11.0) | 185 | (11.4) |
|  | Effective response | 26 | (1.6) | 45 | (5.1) | 36 | (12.4) | 107 | (6.6) |
|  | Lost to follow-up | 4 | (0.2) | 10 | (1.1) | 3 | (1.0) | 17 | (1.1) |
|  | Other ^c^ | 91 | (5.6) | 70 | (7.9) | 18 | (6.2) | 179 | (11.1) |
| **CRC** |  |  |  |  |  |  |  |  |  |
| Total | *N* | 1242 | | 739 | | 229 | | 1242 | |
| Patients who discontinued |  | 503 | (40.5) | 510 | (69.0) | 183 | (79.9) | 1196 | (96.3) |
| Reasons for discontinuation ^b^ | Cancer progression (excluding death) | 155 | (12.5) | 168 | (22.7) | 85 | (37.1) | 408 | (32.9) |
|  | Poor response | 131 | (10.5) | 119 | (16.1) | 23 | (10.0) | 273 | (22.0) |
|  | Adverse events | 103 | (8.3) | 72 | (9.7) | 12 | (5.2) | 187 | (15.1) |
|  | Death | 52 | (4.2) | 62 | (8.4) | 14 | (6.1) | 128 | (10.3) |
|  | Department/hospital transfer | 39 | (3.1) | 50 | (6.8) | 21 | (9.2) | 110 | (8.9) |
|  | Effective response | 26 | (2.1) | 70 | (9.5) | 36 | (15.7) | 132 | (10.6) |
|  | Lost to follow-up | 4 | (0.3) | 1 | (0.1) | 3 | (1.3) | 8 | (0.6) |
|  | Other ^c^ | 82 | (6.6) | 58 | (7.8) | 10 | (4.4) | 150 | (12.1) |

*Note:* Values are *n* (%) of patients.

Abbreviations: CRC, colorectal cancer; GC, gastric cancer; NSCLC, non-small cell lung cancer; PC, pancreatic cancer.

^a^The overall population includes patients with cancers other than NSCLC, GC, PC, and CRC.

^b^Some patients were overlapped.

^c^Other included free description.

**Table S3.** Incidence of treatment-related adverse events listed in the safety specifications

|  | **Overall population ^a^** | | | | **NSCLC** | | | | **GC** | | | | | **PC** | | | | | | **CRC** | | | | | |
| --- | --- | --- | --- | --- | --- | --- | --- | --- | --- | --- | --- | --- | --- | --- | --- | --- | --- | --- | --- | --- | --- | --- | --- | --- | --- |
|  | **(*N* = 6016)** | | | | **(*N* = 1713)** | | | | **(*N* = 1382)** | | | | | **(*N* = 1617)** | | | | | | **(*N* = 1242)** | | | | | |
|  | **Any grade ^b^** | | **Grade ≥3** | | **Any grade ^b^** | | **Grade ≥3** | | **Any grade ^b^** | | **Grade ≥3** | | | **Any grade ^b^** | | | **Grade ≥3** | | | **Any grade ^b^** | | | **Grade ≥3** | | |
| **Hyperglycemia-associated** | 288 | (4.8) | 118 | (2.0) | 82 | (4.8) | 26 | (1.5) | 45 | (3.3) | 16 | (1.2) | 118 | | (7.3) | 61 | | (3.8) | 39 | | (3.1) | 14 | | (1.1) |  |
| Hyperglycemia | 234 | (3.9) | 99 | (1.6) | 60 | (3.5) | 20 | (1.2) | 36 | (2.6) | 12 | (0.9) | 105 | | (6.5) | 55 | | (3.4) | 31 | | (2.5) | 11 | | (0.9) |  |
| Diabetes mellitus | 24 | (0.4) | 7 | (0.1) | 9 | (0.5) | 3 | (0.2) | 4 | (0.3) | - | - | 7 | | (0.4) | 3 | | (0.2) | 4 | | (0.3) | 1 | | (0.1) |  |
| Glycosylated hemoglobin increased | 14 | (0.2) | - | - | 7 | (0.4) | - | - | 1 | (0.1) | - | - | 3 | | (0.2) | - | | - | 1 | | (0.1) | - | | - |  |
| Blood glucose increased | 11 | (0.2) | 5 | (0.1) | 6 | (0.4) | 1 | (0.1) | 2 | (0.1) | 1 | (0.1) | 1 | | (0.1) | 1 | | (0.1) | 2 | | (0.2) | 2 | | (0.2) |  |
| Type 2 diabetes mellitus | 3 | (<0.1) | 2 | (<0.1) | - | - | - | - | 2 | (0.1) | 2 | (0.1) | - | | - | - | | - | 1 | | (0.1) | - | | - |  |
| Diabetic ketoacidosis | 3 | (<0.1) | 3 | (<0.1) | 1 | (0.1) | 1 | (0.1) | 1 | (0.1) | 1 | (0.1) | 1 | | (0.1) | 1 | | (0.1) | - | | - | - | | - |  |
| Glucose tolerance impaired | 1 | (<0.1) | 1 | (<0.1) | 1 | (0.1) | 1 | (0.1) | - | - | - | - | - | | - | - | | - | - | | - | - | | - |  |
| Ketosis | 1 | (<0.1) | - | - | - | - | - | - | - | - | - | - | 1 | | (0.1) | - | | - | - | | - | - | | - |  |
| Pancreatogenous diabetes | 1 | (<0.1) | 1 | (<0.1) | - | - | - | - | - | - | - | - | 1 | | (0.1) | 1 | | (0.1) | - | | - | - | | - |  |
| **Hepatic impairment-associated** | 70 | (1.2) | 16 | (0.3) | 28 | (1.6) | 7 | (0.4) | 15 | (1.1) | 2 | (0.1) | 13 | | (0.8) | 1 | | (0.1) | 14 | | (1.1) | 6 | | (0.5) |  |
| Alanine aminotransferase increased | 21 | (0.3) | 3 | (<0.1) | 12 | (0.7) | 2 | (0.1) | 4 | (0.3) | 1 | (0.1) | 2 | | (0.1) | - | | - | 3 | | (0.2) | - | | - |  |
| Aspartate aminotransferase increased | 20 | (0.3) | 2 | (<0.1) | 10 | (0.6) | 2 | (0.1) | 5 | (0.4) | - | - | 2 | | (0.1) | - | | - | 3 | | (0.2) | - | | - |  |
| Hepatic function abnormal | 17 | (0.3) | 2 | (<0.1) | 8 | (0.5) | 1 | (0.1) | 3 | (0.2) | - | - | 3 | | (0.2) | 1 | | (0.1) | 3 | | (0.2) | - | | - |  |
| Gamma-glutamyltransferase increased | 10 | (0.2) | 2 | (<0.1) | 4 | (0.2) | 1 | (0.1) | 2 | (0.1) | - | - | 2 | | (0.1) | - | | - | 2 | | (0.2) | 1 | | (0.1) |  |
| Liver disorder | 10 | (0.2) | 4 | (0.1) | 2 | (0.1) | 2 | (0.1) | 4 | (0.3) | 1 | (0.1) | 2 | | (0.1) | - | | - | 2 | | (0.2) | 1 | | (0.1) |  |
| Ascites | 3 | (<0.1) | - | - | - | - | - | - | 1 | (0.1) | - | - | 2 | | (0.1) | - | | - | - | | - | - | | - |  |
| Gamma-glutamyltransferase abnormal | 3 | (<0.1) | 1 | (<0.1) | 1 | (0.1) | - | - | - | - | - | - | 1 | | (0.1) | - | | - | 1 | | (0.1) | 1 | | (0.1) |  |
| Blood alkaline phosphatase increased | 2 | (<0.1) | 1 | (<0.1) | 1 | (0.1) | - | - | - | - | - | - | - | | - | - | | - | 1 | | (0.1) | 1 | | (0.1) |  |
| Asterixis | 1 | (<0.1) | - | - | 1 | (0.1) | - | - | - | - | - | - | - | | - | - | | - | - | | - | - | | - |  |
| Blood bilirubin increased | 1 | (<0.1) | - | - | - | - | - | - | - | - | - | - | 1 | | (0.1) | - | | - | - | | - | - | | - |  |
| Hepatic enzyme increased | 1 | (<0.1) | 1 | (<0.1) | 1 | (0.1) | 1 | (0.1) | - | - | - | - | - | | - | - | | - | - | | - | - | | - |  |
| Drug-induced liver injury | 5 | (0.1) | 2 | (<0.1) | 1 | (0.1) | - | - | - | - | - | - | 2 | | (0.1) | - | | - | 2 | | (0.2) | 2 | | (0.2) |  |
| **Conduction disorders-associated** | 65 | (1.1) | 18 | (0.3) | 28 | (1.6) | 7 | (0.4) | 14 | (1.0) | 3 | (0.2) | 10 | | (0.6) | 3 | | (0.2) | 13 | | (1.0) | 5 | | (0.4) |  |
| Electrocardiogram QT prolonged | 15 | (0.2) | 6 | (0.1) | 9 | (0.5) | 6 | (0.4) | 2 | (0.1) | - | - | 1 | | (0.1) | - | | - | 3 | | (0.2) | - | | - |  |
| Sinus tachycardia | 6 | (0.1) | - | - | 2 | (0.1) | - | - | 2 | (0.1) | - | - | 2 | | (0.1) | - | | - | - | | - | - | | - |  |
| Bundle branch block left | 4 | (0.1) | - | - | - | - | - | - | - | - | - | - | 3 | | (0.2) | - | | - | 1 | | (0.1) | - | | - |  |
| Bundle branch block right | 4 | (0.1) | - | - | 1 | (0.1) | - | - | 1 | (0.1) | - | - | - | | - | - | | - | 2 | | (0.2) | - | | - |  |
| Conduction disorder | 4 | (0.1) | - | - | 1 | (0.1) | - | - | 1 | (0.1) | - | - | - | | - | - | | - | 2 | | (0.2) | - | | - |  |
| Ventricular tachycardia | 4 | (0.1) | 4 | (0.1) | - | - | - | - | - | - | - | - | 2 | | (0.1) | 2 | | (0.1) | 2 | | (0.2) | 2 | | (0.2) |  |
| Atrial fibrillation | 3 | (<0.1) | 2 | (<0.1) | 2 | (0.1) | 1 | (0.1) | - | - | - | - | 1 | | (0.1) | 1 | | (0.1) | - | | - | - | | - |  |
| Electrocardiogram QRS complex prolonged | 3 | (<0.1) | - | - | 2 | (0.1) | - | - | - | - | - | - | - | | - | - | | - | 1 | | (0.1) | - | | - |  |
| Atrioventricular block first degree | 3 | (<0.1) | - | - | 2 | (0.1) | - | - | - | - | - | - | 1 | | (0.1) | - | | - | - | | - | - | | - |  |
| Electrocardiogram PR prolongation | 3 | (<0.1) | - | - | 1 | (0.1) | - | - | - | - | - | - | 1 | | (0.1) | - | | - | 1 | | (0.1) | - | | - |  |
| Arrhythmia | 2 | (<0.1) | 1 | (<0.1) | - | - | - | - | 1 | (0.1) | 1 | (0.1) | 1 | | (0.1) | - | | - | - | | - | - | | - |  |
| Long QT syndrome | 2 | (<0.1) | - | - | 1 | (0.1) | - | - | 1 | (0.1) | - | - | - | | - | - | | - | - | | - | - | | - |  |
| Supraventricular extrasystoles | 2 | (<0.1) | - | - | 1 | (0.1) | - | - | 1 | (0.1) | - | - | - | | - | - | | - | - | | - | - | | - |  |
| Ventricular arrhythmia | 2 | (<0.1) | 2 | (<0.1) | - | - | - | - | - | - | - | - | - | | - | - | | - | 2 | | (0.2) | 2 | | (0.2) |  |
| Ventricular extrasystoles | 2 | (<0.1) | - | - | 1 | (0.1) | - | - | 1 | (0.1) | - | - | - | | - | - | | - | - | | - | - | | - |  |
| Atrial flutter | 1 | (<0.1) | - | - | 1 | (0.1) | - | - | - | - | - | - | - | | - | - | | - | - | | - | - | | - |  |
| Atrial tachycardia | 1 | (<0.1) | - | - | 1 | (0.1) | - | - | - | - | - | - | - | | - | - | | - | - | | - | - | | - |  |
| Atrioventricular block second degree | 1 | (<0.1) | - | - | 1 | (0.1) | - | - | - | - | - | - | - | | - | - | | - | - | | - | - | | - |  |
| Defect conduction intraventricular | 1 | (<0.1) | 1 | (<0.1) | - | - | - | - | 1 | (0.1) | 1 | (0.1) | - | | - | - | | - | - | | - | - | | - |  |
| Extrasystoles | 1 | (<0.1) | - | - | - | - | - | - | 1 | (0.1) | - | - | - | | - | - | | - | - | | - | - | | - |  |
| Sinus arrest | 1 | (<0.1) | 1 | (<0.1) | - | - | - | - | 1 | (0.1) | 1 | (0.1) | - | | - | - | | - | - | | - | - | | - |  |
| Sinus arrhythmia | 1 | (<0.1) | - | - | 1 | (0.1) | - | - | - | - | - | - | - | | - | - | | - | - | | - | - | | - |  |
| Sinus bradycardia | 1 | (<0.1) | - | - | - | - | - | - | 1 | (0.1) | - | - | - | | - | - | | - | - | | - | - | | - |  |
| Supraventricular tachycardia | 1 | (<0.1) | - | - | 1 | (0.1) | - | - | - | - | - | - | - | | - | - | | - | - | | - | - | | - |  |
| Tachyarrhythmia | 1 | (<0.1) | - | - | 1 | (0.1) | - | - | - | - | - | - | - | | - | - | | - | - | | - | - | | - |  |
| Bradyarrhythmia | 1 | (<0.1) | 1 | (<0.1) | - | - | - | - | - | - | - | - | - | | - | - | | - | 1 | | (0.1) | 1 | | (0.1) |  |
| Sinus node dysfunction | 1 | (<0.1) | 1 | (<0.1) | - | - | - | - | - | - | - | - | - | | - | - | | - | 1 | | (0.1) | 1 | | (0.1) |  |

*Note:* Values are *n* (%) of patients.

Abbreviations: CRC, colorectal cancer; GC, gastric cancer; NSCLC, non-small cell lung cancer; PC, pancreatic cancer.

^a^The overall population includes patients with cancers other than NSCLC, GC, PC, and CRC.

^b^Treatment-related adverse events with an unknown grade were included.

**Table S4.** Incidence of treatment-related adverse events listed in the safety specifications among patients with the indicated comorbidities

|  |  | **Overall population ^a^** | | | **NSCLC** | | | **GC** | | | **PC** | | | **CRC** | | |
| --- | --- | --- | --- | --- | --- | --- | --- | --- | --- | --- | --- | --- | --- | --- | --- | --- |
|  |  | ***N*** | **Patients with event, *n* (%)** | | ***N*** | **Patients with event, *n* (%)** | | ***N*** | **Patients with event, *n* (%)** | | ***N*** | **Patients with event, *n* (%)** | | ***N*** | **Patients with event, *n* (%)** | |
| **Hyperglycemia-associated** |  |  |  |  |  |  |  |  |  |  |  |  |  |  |  |  |
| Comorbidity:  glucose metabolism disorders | No | 4987 | 131 | (2.6) | 1463 | 35 | (2.4) | 1221 | 25 | (2.0) | 1166 | 48 | (4.1) | 1090 | 22 | (2.0) |
|  | Yes | 1029 | 157 | (15.3) | 250 | 47 | (18.8) | 161 | 20 | (12.4) | 451 | 70 | (15.5) | 152 | 17 | (11.2) |
| **Hepatic impairment-associated** |  |  |  |  |  |  |  |  |  |  |  |  |  |  |  |  |
| Comorbidity:  hepatic disorders | No | 5784 | 61 | (1.1) | 1636 | 23 | (1.4) | 1341 | 14 | (1.0) | 1541 | 12 | (0.8) | 1205 | 12 | (1.0) |
|  | Yes | 232 | 9 | (3.9) | 77 | 5 | (6.5) | 41 | 1 | (2.4) | 76 | 1 | (1.3) | 37 | 2 | (5.4) |
| **Conduction disorders-associated** |  |  |  |  |  |  |  |  |  |  |  |  |  |  |  |  |
| Comorbidity:  heart disorders | No | 5596 | 51 | (0.9) | 1560 | 23 | (1.5) | 1295 | 10 | (0.8) | 1510 | 9 | (0.6) | 1172 | 9 | (0.8) |
|  | Yes | 420 | 14 | (3.3) | 153 | 5 | (3.3) | 87 | 4 | (4.6) | 107 | 1 | (0.9) | 70 | 4 | (5.7) |

Abbreviations: CRC, colorectal cancer; GC, gastric cancer; NSCLC, non-small cell lung cancer; PC, pancreatic cancer.

^a^The overall population includes patients with cancers other than NSCLC, GC, PC, and CRC.

**Table S5.** Incidence rate of the safety specifications by timepoint

|  | **Week 1** | | **Week 2** | | **Week 3** | | **Week 4** | | **Week**  **5–8** | | **Week**  **9–12** | | **Week**  **13–16** | | **Week**  **17–20** | | **Week**  **21–24** | | **Week**  **25–28** | | **Week**  **29–32** | | **Week**  **33–36** | | **Week**  **37–40** | | **Week**  **41–44** | | **Week**  **45–48** | | **Week**  **49–52** | |
| --- | --- | --- | --- | --- | --- | --- | --- | --- | --- | --- | --- | --- | --- | --- | --- | --- | --- | --- | --- | --- | --- | --- | --- | --- | --- | --- | --- | --- | --- | --- | --- | --- |
| **Overall population ^a^** |  |  |  |  |  |  |  |  |  |  |  |  |  |  |  |  |  |  |  |  |  |  |  |  |  |  |  |  |  |  |  |  |
| *N* | 6016 | | 4986 | | 4040 | | 3497 | | 2992 | | 1816 | | 1113 | | 836 | | 658 | | 536 | | 448 | | 370 | | 317 | | 272 | | 236 | | 205 | |
| Hyperglycemia-associated | 100 | (1.7) | 41 | (0.8) | 24 | (0.6) | 30 | (0.9) | 47 | (1.6) | 29 | (1.6) | 14 | (1.3) | 2 | (0.2) | 1 | (0.2) | 2 | (0.4) | - | - | - | - | 1 | (0.3) | - | - | - | - | 1 | (0.5) |
| Hepatic impairment-associated | 30 | (0.5) | 26 | (0.5) | 11 | (0.3) | 6 | (0.2) | 15 | (0.5) | 5 | (0.3) | 1 | (0.1) | - | - | - | - | - | - | - | - | - | - | - | - | - | - | - | - | - | - |
| Conduction disorders-associated | 26 | (0.4) | 13 | (0.3) | 5 | (0.1) | 5 | (0.1) | 8 | (0.3) | 5 | (0.3) | 4 | (0.4) | 1 | (0.1) | 1 | (0.2) | - | - | 2 | (0.4) | 1 | (0.3) | - | - | - | - | - | - | - | - |
| **NSCLC** |  |  |  |  |  |  |  |  |  |  |  |  |  |  |  |  |  |  |  |  |  |  |  |  |  |  |  |  |  |  |  |  |
| *N* | 1713 | | 1446 | | 1190 | | 1045 | | 912 | | 545 | | 311 | | 223 | | 177 | | 149 | | 127 | | 101 | | 87 | | 75 | | 68 | | 61 | |
| Hyperglycemia-associated | 29 | (1.7) | 11 | (0.8) | 7 | (0.6) | 8 | (0.8) | 16 | (1.8) | 11 | (2.0) | 2 | (0.6) | - | - | - | - | - | - | - | - | - | - | - | - | - | - | - | - | - | - |
| Hepatic impairment-associated | 19 | (1.1) | 9 | (0.6) | 5 | (0.4) | 2 | (0.2) | 2 | (0.2) | 4 | (0.7) | - | - | - | - | - | - | - | - | - | - | - | - | - | - | - | - | - | - | - | - |
| Conduction disorders-associated | 11 | (0.6) | 7 | (0.5) | - | - | 2 | (0.2) | 3 | (0.3) | 3 | (0.6) | - | - | - | - | 1 | (0.6) | - | - | 1 | (0.8) | 1 | (1.0) | - | - | - | - | - | - | - | - |
| **GC** |  |  |  |  |  |  |  |  |  |  |  |  |  |  |  |  |  |  |  |  |  |  |  |  |  |  |  |  |  |  |  |  |
| *N* | 1382 | | 1149 | | 927 | | 801 | | 686 | | 428 | | 273 | | 206 | | 161 | | 130 | | 111 | | 86 | | 73 | | 62 | | 54 | | 44 | |
| Hyperglycemia-associated | 12 | (0.9) | 6 | (0.5) | 6 | (0.6) | 4 | (0.5) | 8 | (1.2) | 4 | (0.9) | 5 | (1.8) | - | - | - | - | 1 | (0.8) | - | - | - | - | - | - | - | - | - | - | - | - |
| Hepatic impairment-associated | 1 | (0.1) | 8 | (0.7) | 5 | (0.5) | - | - | 5 | (0.7) | - | - | - | - | - | - | - | - | - | - | - | - | - | - | - | - | - | - | - | - | - | - |
| Conduction disorders-associated | 2 | (0.1) | 3 | (0.3) | 2 | (0.2) | 1 | (0.1) | 2 | (0.3) | - | - | 3 | (1.1) | 1 | (0.5) | - | - | - | - | - | - | - | - | - | - | - | - | - | - | - | - |
| **PC** |  |  |  |  |  |  |  |  |  |  |  |  |  |  |  |  |  |  |  |  |  |  |  |  |  |  |  |  |  |  |  |  |
| *N* | 1617 | | 1298 | | 1047 | | 885 | | 755 | | 465 | | 290 | | 228 | | 177 | | 140 | | 111 | | 101 | | 82 | | 73 | | 58 | | 50 | |
| Hyperglycemia-associated | 52 | (3.2) | 11 | (0.8) | 10 | (1.0) | 13 | (1.5) | 14 | (1.9) | 11 | (2.4) | 6 | (2.1) | 1 | (0.4) | - | - | - | - | - | - | - | - | 1 | (1.2) | - | - | - | - | - | - |
| Hepatic impairment-associated | 6 | (0.4) | 4 | (0.3) | 1 | (0.1) | - | - | 5 | (0.7) | - | - | 1 | (0.3) | - | - | - | - | - | - | - | - | - | - | - | - | - | - | - | - | - | - |
| Conduction disorders-associated | 6 | (0.4) | 2 | (0.2) | 1 | (0.1) | 1 | (0.1) | 1 | (0.1) | - | - | - | - | - | - | - | - | - | - | 1 | (0.9) | - | - | - | - | - | - | - | - | - | - |
| **CRC** |  |  |  |  |  |  |  |  |  |  |  |  |  |  |  |  |  |  |  |  |  |  |  |  |  |  |  |  |  |  |  |  |
| *N* | 1242 | | 1041 | | 842 | | 739 | | 617 | | 362 | | 229 | | 173 | | 137 | | 112 | | 96 | | 81 | | 74 | | 62 | | 56 | | 50 | |
| Hyperglycemia-associated | 7 | (0.6) | 12 | (1.2) | 1 | (0.1) | 4 | (0.5) | 8 | (1.3) | 3 | (0.8) | - | - | 1 | (0.6) | 1 | (0.7) | 1 | (0.9) | - | - | - | - | - | - | - | - | - | - | 1 | (2.0) |
| Hepatic impairment-associated | 4 | (0.3) | 5 | (0.5) | - | - | 4 | (0.5) | 3 | (0.5) | 1 | (0.3) | - | - | - | - | - | - | - | - | - | - | - | - | - | - | - | - | - | - | - | - |
| Conduction disorders-associated | 7 | (0.6) | 1 | (0.1) | 2 | (0.2) | 1 | (0.1) | 2 | (0.3) | 2 | (0.6) | 1 | (0.4) | - | - | - | - | - | - | - | - | - | - | - | - | - | - | - | - | - | - |

*Note:* Values are *n* (%) of patients.

Abbreviations: CRC, colorectal cancer; GC, gastric cancer; NSCLC, non-small cell lung cancer; PC, pancreatic cancer.

^a^The overall population includes patients with cancers other than NSCLC, GC, PC, and CRC.

**Table S6.** Percent changes in body weight according to patient background characteristics

| **Patient background characteristic** | | **Timepoint** | **Overall population** | | | **NSCLC** | | | **GC** | | | **PC** | | | **CRC** | | |
| --- | --- | --- | --- | --- | --- | --- | --- | --- | --- | --- | --- | --- | --- | --- | --- | --- | --- |
|  |  |  | ***N*** | **Mean** | **(SE)** | ***N*** | **Mean** | **(SE)** | ***N*** | **Mean** | **(SE)** | ***N*** | **Mean** | **(SE)** | ***N*** | **Mean** | **(SE)** |
| Age (years) | <75 | Before initiation (kg) ^a^ | 2829 | 50.81 | (0.196) | 739 | 51.03 | (0.387) | 701 | 49.86 | (0.376) | 725 | 50.26 | (0.369) | 654 | 52.18 | (0.439) |
|  |  | Week 3 (% change) ^b^ | 2052 | 1.50 | (0.135) | 500 | 1.91 | (0.276) | 539 | 1.65 | (0.261) | 534 | 1.42 | (0.278) | 470 | 0.99 | (0.271) |
|  |  | Week 12 (% change) ^b^ | 703 | 2.54 | (0.306) | 160 | 3.32 | (0.643) | 195 | 3.16 | (0.601) | 194 | 1.59 | (0.620) | 152 | 2.16 | (0.556) |
|  |  | Week 24 (% change) ^b^ | 266 | 2.98 | (0.555) | 51 | 2.04 | (1.169) | 66 | 3.89 | (1.249) | 83 | 1.93 | (1.000) | 65 | 4.15 | (1.030) |
|  |  | Week 52 (% change) ^b^ | 99 | 2.93 | (0.950) | 20 | 0.50 | (2.098) | 24 | 3.62 | (1.629) | 26 | 2.44 | (2.273) | 29 | 4.47 | (1.589) |
|  | ≥75 | Before initiation (kg) ^a^ | 1636 | 48.36 | (0.228) | 486 | 48.53 | (0.428) | 375 | 48.89 | (0.473) | 446 | 47.12 | (0.419) | 321 | 49.20 | (0.521) |
|  |  | Week 3 (% change) ^b^ | 1123 | 1.56 | (0.176) | 315 | 1.65 | (0.290) | 280 | 0.94 | (0.356) | 296 | 1.82 | (0.373) | 224 | 1.81 | (0.411) |
|  |  | Week 12 (% change) ^b^ | 380 | 3.54 | (0.428) | 113 | 4.54 | (0.671) | 99 | 1.51 | (0.732) | 94 | 4.01 | (0.860) | 70 | 4.12 | (1.313) |
|  |  | Week 24 (% change) ^b^ | 150 | 3.51 | (0.722) | 40 | 4.71 | (0.964) | 41 | 0.67 | (1.107) | 38 | 3.82 | (1.680) | 31 | 5.35 | (2.005) |
|  |  | Week 52 (% change) ^b^ | 61 | 3.91 | (1.374) | 19 | 1.89 | (2.246) | 14 | 1.26 | (2.999) | 13 | 3.74 | (3.124) | 15 | 9.09 | (2.623) |
| ECOG PS | 0–1 | Before initiation (kg) ^a^ | 2661 | 50.81 | (0.191) | 583 | 51.19 | (0.423) | 689 | 50.10 | (0.348) | 775 | 50.05 | (0.347) | 604 | 52.18 | (0.421) |
|  |  | Week 3 (% change) ^b^ | 2058 | 1.33 | (0.119) | 420 | 1.61 | (0.264) | 562 | 1.31 | (0.233) | 581 | 1.46 | (0.233) | 486 | 0.95 | (0.231) |
|  |  | Week 12 (% change) ^b^ | 811 | 2.59 | (0.270) | 166 | 3.58 | (0.542) | 232 | 2.38 | (0.475) | 239 | 2.02 | (0.537) | 169 | 2.75 | (0.620) |
|  |  | Week 24 (% change) ^b^ | 334 | 2.61 | (0.490) | 61 | 3.26 | (0.947) | 91 | 2.30 | (0.934) | 109 | 1.84 | (0.907) | 72 | 3.62 | (1.117) |
|  |  | Week 52 (% change) ^b^ | 124 | 3.26 | (0.895) | 27 | 2.27 | (1.691) | 31 | 2.50 | (1.756) | 34 | 2.21 | (1.947) | 32 | 5.94 | (1.673) |
|  | 2 | Before initiation (kg) ^a^ | 1089 | 49.10 | (0.311) | 374 | 49.27 | (0.513) | 242 | 49.58 | (0.676) | 234 | 46.62 | (0.557) | 234 | 50.91 | (0.768) |
|  |  | Week 3 (% change) ^b^ | 729 | 1.77 | (0.252) | 254 | 1.99 | (0.355) | 169 | 1.77 | (0.542) | 154 | 1.11 | (0.553) | 147 | 2.05 | (0.674) |
|  |  | Week 12 (% change) ^b^ | 204 | 3.59 | (0.638) | 85 | 4.58 | (0.882) | 43 | 4.02 | (1.623) | 35 | 3.18 | (1.834) | 40 | 1.37 | (1.201) |
|  |  | Week 24 (% change) ^b^ | 62 | 4.16 | (1.023) | 26 | 2.67 | (1.576) | 13 | 3.73 | (2.556) | 8 | 7.78 | (3.096) | 15 | 5.21 | (1.701) |
|  |  | Week 52 (% change) ^b^ | 30 | 3.04 | (1.790) | 11 | −0.45 | (3.323) | 6 | 3.93 | (3.010) | 5 | 7.40 | (5.134) | 8 | 4.46 | (3.061) |
|  | 3–4 | Before initiation (kg) ^a^ | 663 | 47.64 | (0.397) | 251 | 48.56 | (0.656) | 132 | 46.76 | (0.912) | 155 | 47.76 | (0.827) | 122 | 46.69 | (0.860) |
|  |  | Week 3 (% change) ^b^ | 351 | 1.98 | (0.406) | 128 | 2.15 | (0.638) | 78 | 1.23 | (0.804) | 93 | 2.63 | (0.946) | 49 | 1.63 | (0.907) |
|  |  | Week 12 (% change) ^b^ | 61 | 4.34 | (1.434) | 19 | 2.11 | (2.737) | 18 | 2.08 | (2.613) | 12 | 7.38 | (1.781) | 12 | 8.21 | (3.868) |
|  |  | Week 24 (% change) ^b^ | 17 | 10.28 | (2.305) | 3 | 3.10 | (3.099) | 2 | 16.90 | (11.500) | 4 | 10.63 | (2.885) | 8 | 11.15 | (3.717) |
|  |  | Week 52 (% change) ^b^ | 6 | 5.52 | (4.692) | 1 | −10.40 | - | 1 | 3.30 | - | 0 | - | - | 4 | 10.05 | (5.164) |
| BMI (kg/m^2^) | <20 | Before initiation (kg) ^a^ | 2662 | 44.75 | (0.134) | 731 | 44.70 | (0.260) | 698 | 45.05 | (0.256) | 732 | 44.58 | (0.254) | 492 | 44.62 | (0.316) |
|  |  | Week 3 (% change) ^b^ | 1931 | 2.20 | (0.145) | 491 | 2.80 | (0.275) | 538 | 1.85 | (0.261) | 524 | 2.26 | (0.294) | 369 | 1.83 | (0.343) |
|  |  | Week 12 (% change) ^b^ | 687 | 4.53 | (0.318) | 182 | 5.46 | (0.574) | 190 | 3.77 | (0.594) | 182 | 4.52 | (0.632) | 130 | 4.32 | (0.798) |
|  |  | Week 24 (% change) ^b^ | 267 | 4.91 | (0.563) | 62 | 4.62 | (0.943) | 73 | 4.22 | (1.108) | 71 | 4.34 | (1.186) | 61 | 6.68 | (1.219) |
|  |  | Week 52 (% change) ^b^ | 99 | 6.05 | (0.920) | 24 | 3.87 | (1.833) | 24 | 5.06 | (1.674) | 22 | 7.08 | (2.120) | 29 | 7.90 | (1.747) |
|  | ≥20 | Before initiation (kg) ^a^ | 1653 | 58.45 | (0.209) | 446 | 59.03 | (0.385) | 352 | 58.68 | (0.439) | 402 | 57.38 | (0.422) | 446 | 58.66 | (0.430) |
|  |  | Week 3 (% change) ^b^ | 1160 | 0.41 | (0.156) | 289 | 0.19 | (0.281) | 266 | 0.69 | (0.353) | 287 | 0.15 | (0.329) | 312 | 0.60 | (0.292) |
|  |  | Week 12 (% change) ^b^ | 381 | −0.10 | (0.364) | 83 | 0.17 | (0.744) | 102 | 0.53 | (0.736) | 103 | −1.46 | (0.717) | 91 | 0.57 | (0.713) |
|  |  | Week 24 (% change) ^b^ | 143 | −0.03 | (0.634) | 26 | −0.54 | (1.349) | 33 | −0.45 | (1.334) | 48 | −0.09 | (1.168) | 35 | 0.80 | (1.289) |
|  |  | Week 52 (% change) ^b^ | 59 | −1.38 | (1.259) | 13 | −4.44 | (2.455) | 14 | −1.21 | (2.626) | 17 | −2.56 | (2.662) | 15 | 2.45 | (2.110) |
| Treatment period | 1st-line | Before initiation (kg) ^a^ | 1267 | 49.71 | (0.268) | 334 | 49.72 | (0.529) | 314 | 50.53 | (0.556) | 415 | 48.75 | (0.452) | 199 | 50.36 | (0.668) |
|  |  | Week 3 (% change) ^b^ | 979 | 1.72 | (0.183) | 233 | 1.78 | (0.366) | 260 | 1.77 | (0.357) | 320 | 1.83 | (0.310) | 161 | 1.25 | (0.486) |
|  |  | Week 12 (% change) ^b^ | 423 | 3.39 | (0.375) | 89 | 4.46 | (0.849) | 120 | 3.30 | (0.756) | 138 | 2.51 | (0.650) | 73 | 3.98 | (0.750) |
|  |  | Week 24 (% change) ^b^ | 202 | 3.94 | (0.634) | 35 | 3.62 | (1.281) | 51 | 3.67 | (1.402) | 70 | 2.67 | (1.024) | 45 | 6.56 | (1.392) |
|  |  | Week 52 (% change) ^b^ | 78 | 5.40 | (1.063) | 19 | 1.11 | (1.676) | 16 | 6.26 | (2.158) | 22 | 3.49 | (2.324) | 21 | 10.63 | (1.683) |
|  | 2nd-line | Before initiation (kg) ^a^ | 794 | 50.77 | (0.346) | 175 | 51.20 | (0.800) | 191 | 50.66 | (0.635) | 239 | 49.71 | (0.598) | 188 | 51.75 | (0.755) |
|  |  | Week 3 (% change) ^b^ | 612 | 1.66 | (0.234) | 128 | 2.64 | (0.489) | 151 | 2.42 | (0.497) | 187 | 1.13 | (0.425) | 145 | 0.71 | (0.451) |
|  |  | Week 12 (% change) ^b^ | 242 | 3.18 | (0.532) | 51 | 4.61 | (0.978) | 66 | 3.00 | (0.944) | 71 | 2.89 | (1.177) | 54 | 2.43 | (1.064) |
|  |  | Week 24 (% change) ^b^ | 89 | 3.39 | (1.056) | 20 | 5.37 | (1.651) | 27 | 1.70 | (1.757) | 22 | 4.51 | (2.858) | 20 | 2.46 | (2.022) |
|  |  | Week 52 (% change) ^b^ | 39 | 0.86 | (1.690) | 9 | 0.47 | (4.138) | 12 | −2.38 | (2.727) | 8 | 5.06 | (4.267) | 10 | 1.74 | (2.833) |
|  | 3rd and later line | Before initiation (kg) ^a^ | 1003 | 50.82 | (0.330) | 300 | 50.19 | (0.614) | 290 | 50.02 | (0.560) | 128 | 50.10 | (0.928) | 282 | 52.70 | (0.654) |
|  |  | Week 3 (% change) ^b^ | 770 | 1.54 | (0.215) | 222 | 1.93 | (0.406) | 235 | 1.03 | (0.353) | 85 | 1.89 | (0.897) | 225 | 1.58 | (0.359) |
|  |  | Week 12 (% change) ^b^ | 247 | 1.89 | (0.450) | 81 | 2.57 | (0.810) | 71 | 1.18 | (0.867) | 31 | 1.58 | (1.329) | 64 | 1.97 | (0.806) |
|  |  | Week 24 (% change) ^b^ | 81 | 1.42 | (0.845) | 27 | 0.46 | (1.378) | 23 | 1.64 | (1.702) | 9 | 0.43 | (2.673) | 22 | 2.78 | (1.635) |
|  |  | Week 52 (% change) ^b^ | 28 | 2.34 | (1.747) | 8 | 0.85 | (4.323) | 8 | 2.81 | (3.060) | 3 | 9.43 | (8.168) | 9 | 0.89 | (1.434) |
|  | BSC | Before initiation (kg) ^a^ | 1138 | 48.44 | (0.305) | 322 | 49.12 | (0.554) | 216 | 46.86 | (0.688) | 320 | 47.77 | (0.539) | 273 | 49.67 | (0.688) |
|  |  | Week 3 (% change) ^b^ | 619 | 1.39 | (0.272) | 166 | 1.96 | (0.461) | 127 | 0.60 | (0.643) | 178 | 1.40 | (0.556) | 142 | 1.44 | (0.542) |
|  |  | Week 12 (% change) ^b^ | 100 | 2.56 | (1.000) | 31 | 4.04 | (1.464) | 17 | 4.03 | (2.736) | 26 | 2.03 | (1.990) | 24 | −0.09 | (2.327) |
|  |  | Week 24 (% change) ^b^ | 23 | 4.33 | (2.097) | 6 | 5.05 | (3.818) | 2 | 7.45 | (1.450) | 9 | 3.46 | (3.309) | 6 | 3.87 | (5.671) |
|  |  | Week 52 (% change) ^b^ | 8 | 6.06 | (3.330) | 3 | 4.67 | (4.757) | 1 | 4.20 | − | 2 | −1.35 | (1.350) | 2 | 16.50 | (8.500) |
| Concomitant therapy | With ICIs | Before initiation (kg) ^a^ | 532 | 50.98 | (0.430) | 314 | 51.59 | (0.571) | 209 | 49.92 | (0.662) | 1 | 44.60 | - | 8 | 55.09 | (3.240) |
|  |  | Week 3 (% change) ^b^ | 435 | 1.79 | (0.268) | 246 | 1.45 | (0.340) | 180 | 2.06 | (0.444) | 1 | 5.40 | - | 8 | 5.58 | (1.173) |
|  |  | Week 12 (% change) ^b^ | 193 | 2.86 | (0.614) | 99 | 3.17 | (0.875) | 90 | 2.33 | (0.890) | 1 | 5.40 | - | 3 | 7.90 | (3.035) |
|  |  | Week 24 (% change) ^b^ | 86 | 2.42 | (0.953) | 34 | 0.29 | (1.255) | 50 | 3.49 | (1.352) | 0 | - | - | 2 | 11.55 | (2.650) |
|  |  | Week 52 (% change) ^b^ | 33 | 1.44 | (1.645) | 15 | 1.51 | (2.101) | 18 | 1.38 | (2.510) | 0 | - | - | 0 | - | - |
|  | Without ICIs | Before initiation (kg) ^a^ | 3933 | 49.77 | (0.161) | 911 | 49.50 | (0.336) | 867 | 49.42 | (0.330) | 1170 | 49.07 | (0.282) | 967 | 51.17 | (0.345) |
|  |  | Week 3 (% change) ^b^ | 2740 | 1.48 | (0.117) | 569 | 1.97 | (0.251) | 639 | 1.22 | (0.239) | 829 | 1.56 | (0.223) | 686 | 1.21 | (0.228) |
|  |  | Week 12 (% change) ^b^ | 890 | 2.89 | (0.272) | 174 | 4.20 | (0.541) | 204 | 2.73 | (0.553) | 287 | 2.37 | (0.508) | 219 | 2.71 | (0.569) |
|  |  | Week 24 (% change) ^b^ | 330 | 3.37 | (0.496) | 57 | 4.95 | (0.948) | 57 | 1.93 | (1.176) | 121 | 2.52 | (0.865) | 94 | 4.39 | (0.961) |
|  |  | Week 52 (% change) ^b^ | 127 | 3.79 | (0.891) | 24 | 0.97 | (2.123) | 20 | 3.98 | (1.746) | 39 | 2.87 | (1.818) | 44 | 6.04 | (1.400) |
|  | With steroids | Before initiation (kg) ^a^ | 769 | 49.33 | (0.358) | 275 | 50.26 | (0.624) | 167 | 49.23 | (0.762) | 167 | 48.60 | (0.703) | 157 | 48.60 | (0.805) |
|  |  | Week 3 (% change) ^b^ | 561 | 1.41 | (0.267) | 189 | 1.55 | (0.481) | 144 | 1.38 | (0.517) | 115 | 1.39 | (0.551) | 110 | 1.23 | (0.620) |
|  |  | Week 12 (% change) ^b^ | 209 | 3.38 | (0.582) | 62 | 3.75 | (1.030) | 66 | 3.17 | (1.037) | 46 | 3.36 | (1.505) | 35 | 3.17 | (1.087) |
|  |  | Week 24 (% change) ^b^ | 81 | 4.09 | (1.148) | 19 | 2.12 | (2.234) | 26 | 4.68 | (2.168) | 22 | 4.00 | (2.121) | 14 | 5.82 | (2.940) |
|  |  | Week 52 (% change) ^b^ | 27 | 2.10 | (2.281) | 7 | -5.07 | (4.638) | 8 | 1.38 | (2.890) | 5 | 7.90 | (6.383) | 7 | 5.97 | (4.444) |
|  | Without steroids | Before initiation (kg) ^a^ | 3696 | 50.03 | (0.166) | 950 | 49.97 | (0.328) | 909 | 49.57 | (0.320) | 1004 | 49.14 | (0.308) | 818 | 51.70 | (0.377) |
|  |  | Week 3 (% change) ^b^ | 2614 | 1.54 | (0.117) | 626 | 1.89 | (0.221) | 675 | 1.41 | (0.230) | 715 | 1.59 | (0.243) | 584 | 1.26 | (0.243) |
|  |  | Week 12 (% change) ^b^ | 874 | 2.77 | (0.275) | 211 | 3.85 | (0.527) | 228 | 2.44 | (0.527) | 242 | 2.20 | (0.532) | 187 | 2.71 | (0.639) |
|  |  | Week 24 (% change) ^b^ | 335 | 2.95 | (0.470) | 72 | 3.50 | (0.811) | 81 | 2.01 | (0.943) | 99 | 2.20 | (0.948) | 82 | 4.32 | (0.996) |
|  |  | Week 52 (% change) ^b^ | 133 | 3.55 | (0.827) | 32 | 2.55 | (1.483) | 30 | 3.12 | (1.750) | 34 | 2.14 | (1.873) | 37 | 6.06 | (1.470) |
| History of gastric surgery | No history of gastric surgery | Before initiation (kg) ^a^ | 3713 | 50.39 | (0.166) | 1188 | 50.15 | (0.294) | 504 | 51.63 | (0.423) | 1069 | 49.21 | (0.293) | 939 | 51.34 | (0.350) |
|  |  | Week 3 (% change) ^b^ | 2603 | 1.45 | (0.119) | 788 | 1.82 | (0.206) | 378 | 0.90 | (0.327) | 757 | 1.51 | (0.228) | 668 | 1.29 | (0.233) |
|  |  | Week 12 (% change) ^b^ | 885 | 2.69 | (0.269) | 265 | 3.67 | (0.476) | 138 | 1.93 | (0.689) | 266 | 2.37 | (0.530) | 212 | 2.40 | (0.509) |
|  |  | Week 24 (% change) ^b^ | 335 | 3.37 | (0.500) | 87 | 3.11 | (0.817) | 44 | 3.27 | (1.463) | 111 | 2.52 | (0.934) | 92 | 4.71 | (0.975) |
|  |  | Week 52 (% change) ^b^ | 131 | 3.09 | (0.916) | 37 | 1.07 | (1.595) | 15 | −0.15 | (2.781) | 35 | 2.90 | (2.026) | 44 | 6.04 | (1.400) |
|  | Total gastrectomy | Before initiation (kg) ^a^ | 261 | 45.31 | (0.540) | 6 | 38.53 | (1.606) | 230 | 45.67 | (0.577) | 18 | 44.17 | (2.270) | 6 | 40.57 | (1.468) |
|  |  | Week 3 (% change) ^b^ | 202 | 1.77 | (0.399) | 5 | 2.54 | (1.349) | 178 | 1.68 | (0.435) | 12 | 3.08 | (1.302) | 6 | −0.35 | (1.739) |
|  |  | Week 12 (% change) ^b^ | 72 | 3.65 | (0.965) | 2 | 4.95 | (2.550) | 64 | 3.03 | (1.013) | 2 | 11.80 | (8.500) | 4 | 8.88 | (4.080) |
|  |  | Week 24 (% change) ^b^ | 30 | 3.09 | (1.654) | 1 | 3.60 | - | 26 | 2.80 | (1.823) | 1 | 14.20 | - | 2 | 1.00 | (6.500) |
|  |  | Week 52 (% change) ^b^ | 11 | 2.82 | (2.003) | 1 | 0.00 | - | 10 | 3.10 | (2.193) | 0 | - | - | 0 | - | - |
|  | Partial gastrectomy (proximal or distal) | Before initiation (kg) ^a^ | 295 | 47.87 | (0.578) | 15 | 47.23 | (2.908) | 243 | 48.34 | (0.632) | 21 | 43.40 | (1.987) | 14 | 47.78 | (2.877) |
|  |  | Week 3 (% change) ^b^ | 223 | 2.26 | (0.345) | 12 | 1.66 | (1.357) | 188 | 2.44 | (0.384) | 14 | 1.08 | (1.301) | 7 | 1.29 | (1.407) |
|  |  | Week 12 (% change) ^b^ | 86 | 4.31 | (0.822) | 6 | 10.23 | (2.754) | 70 | 3.91 | (0.925) | 6 | 2.67 | (1.856) | 3 | 4.87 | (6.286) |
|  |  | Week 24 (% change) ^b^ | 38 | 1.53 | (1.389) | 3 | 5.87 | (3.597) | 30 | 1.02 | (1.700) | 4 | 1.03 | (1.730) | 1 | 5.90 | - |
|  |  | Week 52 (% change) ^b^ | 13 | 5.46 | (1.698) | 1 | 6.50 | - | 10 | 5.88 | (2.194) | 2 | 2.85 | (0.250) | 0 | - | - |

Abbreviations: BMI, body mass index; BSC, best supportive care; CRC, colorectal cancer; ECOG PS, Eastern Cooperative Oncology Group Performance Status; GC, gastric cancer; ICIs, immune checkpoint inhibitors; NSCLC, non-small cell lung cancer; PC, pancreatic cancer; SE, standard error.

^a^Baseline value before start of anamorelin treatment.

^b^Percent change from baseline.

**Table S7.** Changes in FAACT-5IASS total scores according to patient background characteristics

| **Patient background characteristic** | | **Timepoint** | **Overall population** | | | **NSCLC** | | | **GC** | | | **PC** | | | **CRC** | | | |
| --- | --- | --- | --- | --- | --- | --- | --- | --- | --- | --- | --- | --- | --- | --- | --- | --- | --- | --- |
|  |  |  | ***N*** | **Mean** | **(SE)** | ***N*** | **Mean** | **(SE)** | ***N*** | **Mean** | **(SE)** | ***N*** | **Mean** | **(SE)** | ***N*** | **Mean** | **(SE)** |  |
| Age (years) | <75 | Before initiation (value) ^a^ | 2359 | 8.0 | (0.09) | 608 | 7.5 | (0.17) | 594 | 8.0 | (0.17) | 605 | 8.2 | (0.17) | 543 | 8.2 | (0.18) |  |
|  |  | Week 3 (change) ^b^ | 1936 | 3.3 | (0.11) | 497 | 4.2 | (0.24) | 488 | 3.1 | (0.21) | 501 | 2.7 | (0.20) | 443 | 3.2 | (0.22) |  |
|  |  | Week 12 (change) ^b^ | 617 | 4.6 | (0.21) | 155 | 5.6 | (0.40) | 163 | 4.5 | (0.40) | 166 | 3.2 | (0.42) | 131 | 5.2 | (0.47) |  |
|  |  | Week 24 (change) ^b^ | 211 | 4.9 | (0.39) | 48 | 6.6 | (0.83) | 46 | 4.8 | (0.80) | 64 | 3.4 | (0.68) | 53 | 5.2 | (0.78) |  |
|  |  | Week 52 (change) ^b^ | 77 | 4.8 | (0.59) | 14 | 5.1 | (1.50) | 19 | 3.8 | (1.12) | 21 | 4.1 | (1.27) | 23 | 6.0 | (0.98) |  |
|  | ≥75 | Before initiation (value) ^a^ | 1433 | 7.9 | (0.12) | 416 | 8.0 | (0.23) | 333 | 8.0 | (0.25) | 400 | 7.9 | (0.23) | 278 | 7.8 | (0.26) |  |
|  |  | Week 3 (change) ^b^ | 1212 | 3.0 | (0.14) | 356 | 3.3 | (0.28) | 283 | 2.6 | (0.30) | 327 | 2.7 | (0.27) | 241 | 3.5 | (0.31) |  |
|  |  | Week 12 (change) ^b^ | 340 | 5.0 | (0.31) | 103 | 5.6 | (0.59) | 85 | 4.8 | (0.68) | 89 | 4.1 | (0.58) | 61 | 5.8 | (0.66) |  |
|  |  | Week 24 (change) ^b^ | 135 | 5.7 | (0.45) | 39 | 7.4 | (0.91) | 34 | 5.6 | (0.82) | 36 | 4.2 | (0.82) | 25 | 5.7 | (1.02) |  |
|  |  | Week 52 (change) ^b^ | 48 | 6.1 | (0.76) | 16 | 8.5 | (1.36) | 10 | 5.3 | (1.71) | 11 | 2.8 | (1.44) | 11 | 6.6 | (1.08) |  |
| ECOG PS | 0–1 | Before initiation (value) ^a^ | 2234 | 8.6 | (0.09) | 479 | 8.5 | (0.20) | 589 | 8.4 | (0.17) | 656 | 8.7 | (0.16) | 501 | 8.9 | (0.19) |  |
|  |  | Week 3 (change) ^b^ | 1888 | 3.3 | (0.11) | 405 | 3.9 | (0.26) | 497 | 3.3 | (0.22) | 550 | 2.8 | (0.19) | 429 | 3.4 | (0.21) |  |
|  |  | Week 12 (change) ^b^ | 703 | 4.6 | (0.20) | 156 | 5.8 | (0.43) | 193 | 4.4 | (0.39) | 209 | 3.3 | (0.37) | 141 | 5.5 | (0.44) |  |
|  |  | Week 24 (change) ^b^ | 267 | 4.9 | (0.34) | 57 | 7.2 | (0.74) | 66 | 5.2 | (0.66) | 87 | 3.1 | (0.55) | 56 | 5.1 | (0.73) |  |
|  |  | Week 52 (change)^b^ | 98 | 5.0 | (0.53) | 20 | 5.8 | (1.19) | 24 | 4.3 | (1.10) | 29 | 3.3 | (1.02) | 25 | 6.8 | (0.85) |  |
|  | 2 | Before initiation (value) ^a^ | 924 | 7.3 | (0.13) | 312 | 7.2 | (0.24) | 205 | 7.7 | (0.29) | 205 | 7.5 | (0.29) | 198 | 7.1 | (0.28) |  |
|  |  | Week 3 (change) ^b^ | 760 | 3.0 | (0.18) | 265 | 3.9 | (0.33) | 166 | 2.3 | (0.33) | 164 | 2.2 | (0.34) | 162 | 3.0 | (0.38) |  |
|  |  | Week 12 (change) ^b^ | 181 | 5.1 | (0.39) | 77 | 5.5 | (0.58) | 34 | 5.3 | (0.88) | 33 | 4.2 | (1.03) | 37 | 4.6 | (0.85) |  |
|  |  | Week 24 (change) ^b^ | 55 | 5.9 | (0.67) | 24 | 7.0 | (1.24) | 11 | 5.6 | (1.23) | 8 | 6.5 | (0.85) | 12 | 3.8 | (1.14) |  |
|  |  | Week 52 (change) ^b^ | 22 | 6.3 | (1.11) | 9 | 9.0 | (2.06) | 4 | 5.3 | (1.25) | 3 | 6.7 | (2.60) | 6 | 2.8 | (1.40) |  |
|  | 3–4 | Before initiation (value) ^a^ | 611 | 6.4 | (0.18) | 227 | 6.7 | (0.30) | 124 | 6.2 | (0.41) | 141 | 6.0 | (0.36) | 117 | 6.6 | (0.43) |  |
|  |  | Week 3 (change) ^b^ | 477 | 3.0 | (0.24) | 177 | 3.5 | (0.41) | 99 | 2.0 | (0.48) | 111 | 2.9 | (0.51) | 88 | 3.2 | (0.60) |  |
|  |  | Week 12 (change) ^b^ | 66 | 5.7 | (0.83) | 22 | 5.0 | (1.39) | 20 | 5.3 | (1.71) | 12 | 5.8 | (1.56) | 12 | 7.6 | (2.05) |  |
|  |  | Week 24 (change) ^b^ | 21 | 8.5 | (1.26) | 4 | 7.8 | (3.28) | 3 | 2.0 | (0.58) | 5 | 9.6 | (3.11) | 9 | 10.3 | (1.59) |  |
|  |  | Week 52 (change) ^b^ | 5 | 7.2 | (2.27) | 1 | 12.0 | - | 1 | 1.0 | - | 0 | - | - | 3 | 7.7 | (2.60) |  |
| BMI (kg/m^2^) | <20 | Before initiation (value) ^a^ | 2282 | 7.9 | (0.09) | 615 | 7.7 | (0.18) | 604 | 7.9 | (0.17) | 636 | 8.0 | (0.17) | 419 | 8.0 | (0.22) |  |
|  |  | Week 3 (change) ^b^ | 1909 | 3.3 | (0.11) | 514 | 3.9 | (0.23) | 502 | 3.0 | (0.20) | 530 | 2.8 | (0.21) | 357 | 3.3 | (0.25) |  |
|  |  | Week 12 (change) ^b^ | 605 | 5.1 | (0.21) | 166 | 5.7 | (0.41) | 165 | 4.8 | (0.41) | 164 | 4.1 | (0.40) | 107 | 5.9 | (0.51) |  |
|  |  | Week 24 (change) ^b^ | 218 | 5.3 | (0.35) | 54 | 7.2 | (0.70) | 54 | 5.6 | (0.62) | 62 | 3.1 | (0.61) | 47 | 5.8 | (0.79) |  |
|  |  | Week 52 (change) ^b^ | 76 | 5.6 | (0.59) | 16 | 8.3 | (1.09) | 19 | 4.5 | (1.35) | 17 | 3.8 | (1.36) | 24 | 6.0 | (0.86) |  |
|  | ≥20 | Before initiation (value) ^a^ | 1338 | 8.1 | (0.12) | 358 | 7.6 | (0.23) | 292 | 8.4 | (0.25) | 323 | 8.3 | (0.24) | 359 | 8.3 | (0.22) |  |
|  |  | Week 3 (change) ^b^ | 1090 | 3.2 | (0.15) | 294 | 3.8 | (0.33) | 246 | 2.9 | (0.32) | 257 | 2.4 | (0.27) | 288 | 3.2 | (0.27) |  |
|  |  | Week 12 (change) ^b^ | 328 | 4.4 | (0.32) | 79 | 5.9 | (0.64) | 82 | 4.2 | (0.67) | 84 | 2.4 | (0.64) | 82 | 4.9 | (0.59) |  |
|  |  | Week 24 (change) ^b^ | 119 | 5.2 | (0.55) | 29 | 7.6 | (1.15) | 26 | 4.2 | (1.21) | 33 | 4.2 | (0.99) | 31 | 4.7 | (1.00) |  |
|  |  | Week 52 (change) ^b^ | 46 | 5.1 | (0.78) | 12 | 6.5 | (1.94) | 10 | 3.9 | (0.96) | 14 | 3.6 | (1.49) | 10 | 6.5 | (1.50) |  |
| Treatment period | 1st-line | Before initiation (value) ^a^ | 1057 | 8.3 | (0.13) | 286 | 7.7 | (0.27) | 269 | 8.7 | (0.26) | 336 | 8.3 | (0.23) | 161 | 8.8 | (0.33) |  |
|  |  | Week 3 (change) ^b^ | 875 | 3.8 | (0.17) | 237 | 4.6 | (0.37) | 225 | 3.6 | (0.33) | 274 | 3.1 | (0.28) | 135 | 4.3 | (0.41) |  |
|  |  | Week 12 (change) ^b^ | 363 | 5.1 | (0.29) | 83 | 6.1 | (0.62) | 98 | 4.8 | (0.52) | 116 | 4.3 | (0.52) | 63 | 5.8 | (0.68) |  |
|  |  | Week 24 (change) ^b^ | 160 | 5.1 | (0.43) | 35 | 7.0 | (0.95) | 38 | 4.7 | (0.76) | 53 | 3.8 | (0.74) | 34 | 5.7 | (1.00) |  |
|  |  | Week 52 (change) ^b^ | 63 | 5.7 | (0.63) | 16 | 7.0 | (1.44) | 16 | 5.0 | (1.05) | 15 | 4.0 | (1.41) | 16 | 6.6 | (1.09) |  |
|  | 2nd-line | Before initiation (value) ^a^ | 654 | 8.2 | (0.17) | 136 | 7.4 | (0.37) | 168 | 8.2 | (0.33) | 199 | 8.8 | (0.30) | 150 | 8.3 | (0.36) |  |
|  |  | Week 3 (change) ^b^ | 553 | 3.2 | (0.20) | 117 | 4.1 | (0.45) | 138 | 3.1 | (0.40) | 169 | 2.6 | (0.35) | 128 | 3.3 | (0.36) |  |
|  |  | Week 12 (change) ^b^ | 205 | 4.7 | (0.37) | 46 | 5.0 | (0.88) | 56 | 4.3 | (0.73) | 58 | 3.8 | (0.66) | 45 | 5.9 | (0.71) |  |
|  |  | Week 24 (change) ^b^ | 80 | 5.4 | (0.54) | 20 | 7.7 | (1.26) | 22 | 4.2 | (0.92) | 18 | 3.8 | (0.79) | 20 | 6.0 | (1.13) |  |
|  |  | Week 52 (change) ^b^ | 33 | 5.4 | (0.95) | 9 | 8.1 | (1.95) | 9 | 1.8 | (1.35) | 7 | 4.0 | (2.07) | 8 | 7.6 | (1.50) |  |
|  | 3rd and later line | Before initiation (value) ^a^ | 814 | 8.1 | (0.15) | 232 | 7.8 | (0.28) | 244 | 8.1 | (0.26) | 112 | 8.0 | (0.39) | 223 | 8.5 | (0.28) |  |
|  |  | Week 3 (change) ^b^ | 694 | 3.1 | (0.17) | 197 | 3.7 | (0.36) | 210 | 2.7 | (0.31) | 92 | 3.0 | (0.45) | 192 | 3.0 | (0.31) |  |
|  |  | Week 12 (change) ^b^ | 210 | 4.4 | (0.35) | 72 | 5.1 | (0.57) | 60 | 3.8 | (0.71) | 29 | 2.8 | (0.78) | 49 | 4.9 | (0.76) |  |
|  |  | Week 24 (change) ^b^ | 58 | 5.3 | (0.78) | 21 | 5.6 | (1.34) | 14 | 6.6 | (1.80) | 9 | 4.6 | (1.43) | 14 | 3.9 | (1.64) |  |
|  |  | Week 52 (change) ^b^ | 16 | 4.4 | (1.07) | 2 | 7.0 | (4.00) | 3 | 3.3 | (2.03) | 4 | 4.3 | (2.66) | 7 | 4.1 | (1.65) |  |
|  | BSC | Before initiation (value) ^a^ | 1056 | 7.2 | (0.13) | 293 | 7.4 | (0.25) | 199 | 6.5 | (0.32) | 300 | 7.4 | (0.25) | 258 | 7.3 | (0.27) |  |
|  |  | Week 3 (change) ^b^ | 847 | 2.6 | (0.17) | 238 | 3.1 | (0.33) | 159 | 2.2 | (0.37) | 240 | 2.2 | (0.31) | 206 | 2.9 | (0.36) |  |
|  |  | Week 12 (change) ^b^ | 121 | 4.3 | (0.59) | 40 | 5.3 | (0.88) | 19 | 6.4 | (1.93) | 32 | 1.7 | (1.15) | 29 | 4.1 | (1.07) |  |
|  |  | Week 24 (change) ^b^ | 33 | 5.7 | (1.01) | 9 | 8.3 | (2.03) | 4 | 8.5 | (3.71) | 11 | 3.4 | (1.66) | 8 | 5.4 | (1.44) |  |
|  |  | Week 52 (change) ^b^ | 8 | 6.9 | (2.12) | 2 | 5.5 | (4.50) | 1 | 19.0 | - | 3 | 3.3 | (1.86) | 2 | 7.5 | (0.50) |  |
| Concomitant therapy | With ICIs | Before initiation (value) ^a^ | 436 | 8.1 | (0.21) | 251 | 7.9 | (0.29) | 180 | 8.3 | (0.32) | 0 | - | - | 5 | 9.2 | (2.62) |  |
|  |  | Week 3 (change) ^b^ | 387 | 3.9 | (0.26) | 221 | 4.1 | (0.36) | 161 | 3.4 | (0.38) | 0 | - | - | 5 | 4.8 | (2.84) |  |
|  |  | Week 12 (change) ^b^ | 173 | 4.8 | (0.41) | 93 | 4.9 | (0.59) | 78 | 4.7 | (0.59) | 0 | - | - | 2 | 8.0 | (4.00) |  |
|  |  | Week 24 (change) ^b^ | 67 | 6.1 | (0.63) | 30 | 7.8 | (0.90) | 36 | 4.5 | (0.82) | 0 | - | - | 1 | 12.0 | - |  |
|  |  | Week 52 (change) ^b^ | 25 | 5.1 | (0.99) | 12 | 6.7 | (1.55) | 13 | 3.6 | (1.17) | 0 | - | - | 0 | - | - |  |
|  | Without ICIs | Before initiation (value) ^a^ | 3356 | 7.9 | (0.07) | 773 | 7.6 | (0.16) | 747 | 7.9 | (0.16) | 1005 | 8.1 | (0.14) | 816 | 8.1 | (0.15) |  |
|  |  | Week 3 (change) ^b^ | 2761 | 3.1 | (0.09) | 632 | 3.7 | (0.21) | 610 | 2.8 | (0.19) | 828 | 2.7 | (0.16) | 679 | 3.3 | (0.18) |  |
|  |  | Week 12 (change) ^b^ | 784 | 4.7 | (0.20) | 165 | 6.0 | (0.41) | 170 | 4.6 | (0.44) | 255 | 3.5 | (0.34) | 190 | 5.4 | (0.39) |  |
|  |  | Week 24 (change) ^b^ | 279 | 5.0 | (0.33) | 57 | 6.6 | (0.81) | 44 | 5.7 | (0.80) | 100 | 3.7 | (0.53) | 77 | 5.3 | (0.62) |  |
|  |  | Week 52 (change) ^b^ | 100 | 5.3 | (0.53) | 18 | 7.1 | (1.42) | 16 | 4.9 | (1.41) | 32 | 3.7 | (0.96) | 34 | 6.2 | (0.74) |  |
|  | With steroids | Before initiation (value) ^a^ | 611 | 7.8 | (0.19) | 223 | 7.5 | (0.32) | 127 | 8.4 | (0.41) | 135 | 8.1 | (0.36) | 124 | 7.4 | (0.41) |  |
|  |  | Week 3 (change) ^b^ | 519 | 3.6 | (0.23) | 184 | 4.1 | (0.44) | 111 | 3.4 | (0.47) | 119 | 2.4 | (0.41) | 104 | 4.1 | (0.50) |  |
|  |  | Week 12 (change) ^b^ | 178 | 4.9 | (0.43) | 57 | 6.1 | (0.81) | 51 | 4.0 | (0.74) | 42 | 3.7 | (1.00) | 28 | 5.7 | (0.81) |  |
|  |  | Week 24 (change) ^b^ | 58 | 4.7 | (0.68) | 18 | 5.9 | (1.49) | 15 | 4.6 | (1.11) | 16 | 4.0 | (1.30) | 9 | 4.1 | (1.46) |  |
|  |  | Week 52 (change) ^b^ | 21 | 4.1 | (1.31) | 4 | 1.8 | (3.52) | 7 | 3.4 | (2.08) | 4 | 4.5 | (4.65) | 6 | 6.3 | (1.61) |  |
|  | Without steroids | Before initiation (value) ^a^ | 3181 | 8.0 | (0.08) | 801 | 7.7 | (0.15) | 800 | 7.9 | (0.15) | 870 | 8.1 | (0.15) | 697 | 8.2 | (0.16) |  |
|  |  | Week 3 (change) ^b^ | 2629 | 3.1 | (0.09) | 669 | 3.8 | (0.20) | 660 | 2.8 | (0.18) | 709 | 2.7 | (0.17) | 580 | 3.1 | (0.19) |  |
|  |  | Week 12 (change) ^b^ | 779 | 4.7 | (0.19) | 201 | 5.4 | (0.37) | 197 | 4.8 | (0.40) | 213 | 3.5 | (0.36) | 164 | 5.3 | (0.43) |  |
|  |  | Week 24 (change) ^b^ | 288 | 5.3 | (0.33) | 69 | 7.3 | (0.67) | 65 | 5.3 | (0.66) | 84 | 3.6 | (0.58) | 69 | 5.6 | (0.67) |  |
|  |  | Week 52 (change) ^b^ | 104 | 5.5 | (0.50) | 26 | 7.7 | (1.01) | 22 | 4.6 | (1.06) | 28 | 3.5 | (0.93) | 28 | 6.1 | (0.84) |  |
| History of gastric surgery | No history of gastric surgery | Before initiation (value) ^a^ | 3130 | 7.9 | (0.08) | 991 | 7.7 | (0.14) | 420 | 8.0 | (0.20) | 915 | 8.1 | (0.14) | 793 | 8.1 | (0.15) |  |
|  |  | Week 3 (change) ^b^ | 2589 | 3.2 | (0.10) | 822 | 3.8 | (0.19) | 346 | 2.8 | (0.25) | 751 | 2.7 | (0.17) | 660 | 3.3 | (0.18) |  |
|  |  | Week 12 (change) ^b^ | 786 | 4.7 | (0.19) | 250 | 5.5 | (0.34) | 111 | 3.6 | (0.47) | 236 | 3.5 | (0.36) | 186 | 5.5 | (0.39) |  |
|  |  | Week 24 (change) ^b^ | 287 | 5.3 | (0.33) | 84 | 6.9 | (0.63) | 35 | 5.2 | (0.86) | 92 | 3.7 | (0.56) | 76 | 5.5 | (0.63) |  |
|  |  | Week 52 (change) ^b^ | 102 | 5.5 | (0.52) | 29 | 6.9 | (1.07) | 11 | 4.5 | (1.25) | 28 | 3.6 | (1.08) | 34 | 6.2 | (0.74) |  |
|  | Total gastrectomy | Before initiation (value) ^a^ | 228 | 8.0 | (0.29) | 6 | 4.4 | (1.83) | 200 | 8.1 | (0.31) | 16 | 8.7 | (1.24) | 5 | 9.2 | (1.66) |  |
|  |  | Week 3 (change) ^b^ | 188 | 2.9 | (0.34) | 5 | 6.9 | (3.55) | 164 | 2.9 | (0.36) | 14 | 2.1 | (1.29) | 5 | 0.6 | (0.81) |  |
|  |  | Week 12 (change) ^b^ | 64 | 4.6 | (0.64) | 3 | 3.6 | (1.56) | 57 | 4.7 | (0.72) | 2 | 5.0 | (0.00) | 2 | 3.5 | (0.50) |  |
|  |  | Week 24 (change) ^b^ | 21 | 4.5 | (0.81) | 1 | 9.0 | - | 18 | 4.7 | (0.85) | 1 | 2.0 | - | 1 | –1.0 | - |  |
|  |  | Week 52 (change) ^b^ | 8 | 4.4 | (1.99) | 1 | 9.0 | - | 7 | 3.7 | (2.17) | 0 | - | - | 0 | - | - |  |
|  | Partial gastrectomy (proximal or distal) | Before initiation (value) ^a^ | 260 | 8.2 | (0.28) | 12 | 6.3 | (0.97) | 219 | 8.1 | (0.31) | 17 | 8.1 | (1.24) | 11 | 10.9 | (1.18) |  |
|  |  | Week 3 (change) ^b^ | 222 | 3.2 | (0.35) | 11 | 2.9 | (1.44) | 189 | 3.2 | (0.38) | 15 | 3.2 | (1.36) | 7 | 3.6 | (1.88) |  |
|  |  | Week 12 (change) ^b^ | 72 | 5.8 | (0.70) | 4 | 11.3 | (2.14) | 60 | 6.0 | (0.77) | 5 | 3.2 | (1.77) | 3 | -0.3 | (1.20) |  |
|  |  | Week 24 (change) ^b^ | 26 | 5.8 | (0.97) | 2 | 8.0 | (3.00) | 19 | 5.9 | (1.24) | 4 | 4.8 | (1.93) | 1 | 2.0 | - |  |
|  |  | Week 52 (change) ^b^ | 10 | 6.1 | (1.57) | 0 | - | - | 8 | 6.0 | (1.98) | 2 | 6.5 | (0.50) | 0 | - | - |  |

Abbreviations: BMI, body mass index; BSC, best supportive care; CRC, colorectal cancer; ECOG PS, Eastern Cooperative Oncology Group Performance Status; GC, gastric cancer; ICIs, immune checkpoint inhibitors; FAACT-5IASS, Functional Assessment of Anorexia/Cachexia Therapy 5-item Anorexia Symptom Scale; NSCLC, non-small cell lung cancer; PC, pancreatic cancer; SE, standard error.

^a^Baseline value before start of anamorelin treatment.

^b^Change from baseline.

**Figure S1.** Changes in food intake relative to the baseline in the overall population (A), and in patients with NSCLC (B), GC (C), PC (D), or CRC (E)


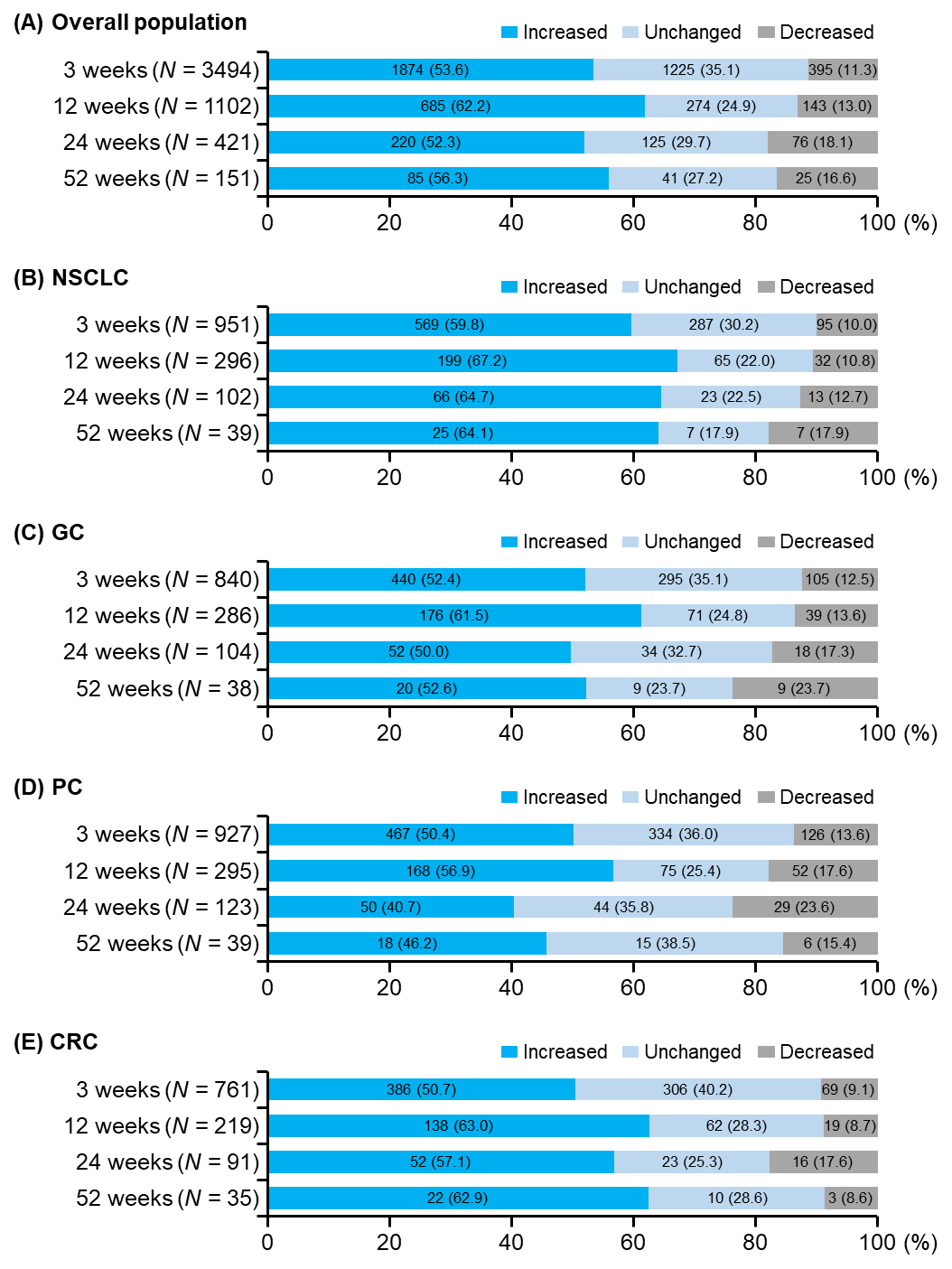


Values are *n* (%) of patients.

Abbreviations: CRC, colorectal cancer; GC, gastric cancer; NSCLC, non-small cell lung cancer; PC, pancreatic cancer.
